# Based on cuproptosis-related lncRNAs, a novel prognostic signature for colon adenocarcinoma prognosis, immunotherapy, and chemotherapy response

**DOI:** 10.3389/fphar.2023.1200054

**Published:** 2023-06-12

**Authors:** Chong Li, Keqian Zhang, Yuzhu Gong, Qinan Wu, Yanyan Zhang, Yan Dong, Dejia Li, Zhe Wang

**Affiliations:** ^1^ Department of Occupational and Environmental Health, School of Public Health, Wuhan University, Wuhan, China; ^2^ Department of Oncology, Dazu Hospital of Chongqing Medical University, Chongqing, China; ^3^ Department of Oncology and Southwest Cancer Center, Southwest Hospital, Third Military Medical University (Army Medical University), Chongqing, China; ^4^ Endocrinology Department, Dazu Hospital of Chongqing Medical University, Chongqing, China

**Keywords:** TCGA, colon adenocarcinoma, cuproptosis, lncRNA, prognostic, immunotherapy response

## Abstract

**Introduction:** Colon adenocarcinoma (COAD) is a special pathological subtype of colorectal cancer (CRC) with highly heterogeneous solid tumors with poor prognosis, and novel biomarkers are urgently required to guide its prognosis.

**Material and methods:** RNA-Seq data of COAD were downloaded through The Cancer Genome Atlas (TCGA) database to determine cuproptosis-related lncRNAs (CRLs) using weighted gene co-expression network analysis (WGCNA). The scores of the pathways were calculated by single-sample gene set enrichment analysis (ssGSEA). CRLs that affected prognoses were determined via the univariate COX regression analysis to develop a prognostic model using multivariate COX regression analysis and LASSO regression analysis. The model was assessed by applying Kaplan–Meier (K-M) survival analysis and receiver operating characteristic curves and validated in GSE39582 and GSE17538. The tumor microenvironment (TME), single nucleotide variants (SNV), and immunotherapy response/chemotherapy sensitivity were assessed in high- and low-score subgroups. Finally, the construction of a nomogram was adopted to predict survival rates of COAD patients during years 1, 3, and 5.

**Results:** We found that a high cuproptosis score reduced the survival rates of COAD significantly. A total of five CRLs affecting prognosis were identified, containing AC008494.3, EIF3J-DT, AC016027.1, AL731533.2, and ZEB1-AS1. The ROC curve showed that RiskScore could perform well in predicting the prognosis of COAD. Meanwhile, we found that RiskScore showed good ability in assessing immunotherapy and chemotherapy sensitivity. Finally, the nomogram and decision curves showed that RiskScore would be a powerful predictor for COAD.

**Conclusion:** A novel prognostic model was constructed using CRLs in COAD, and the CRLs in the model were probably a potential therapeutic target. Based on this study, RiskScore was an independent predictor factor, immunotherapy response, and chemotherapy sensitivity for COAD, providing a new scientific basis for COAD prognosis management.

## Introduction

Colorectal cancer (CRC) is the third most frequent malignant solid tumor among young populations, with the third highest mortality rate among solid tumors (Miller et al., 2020). CRC has shown a high degree of heterogeneity, even though the same type of tumor often exhibited different biological behavior in different populations (Punt et al., 2017). Colonic adenocarcinoma (COAD), the most frequent histological subtype of CRC, largely occurs in the intestinal mucosa and is highly aggressive, leading to high morbidity and mortality (Benson et al., 2018; Ferlay et al., 2021). Due to the absence of signature early symptoms, most patients are already metastatic when diagnosed, which leads to a lower 5-year survival rate of only 14% even with systemic therapy (Siegel et al., 2022). As the existing prognostic indicators for COAD cannot reveal its biological heterogeneity, it is crucial to tap more accurate predictive tools that can combine clinicopathological and molecular characteristics (Yang et al., 2022).

Currently, Tsvetkov et al. (2022) proposed a newly programmed cell death regulated through accumulating copper ions known as cuproptosis, which differs from foregone cell death mechanisms such as apoptosis, autophagy, and ferroptosis. It has been found that copper can still induce cell death when known cell death mechanisms become blocked. During mitochondrial respiration in eukaryotic cells, the lipid acylation component of the tricarboxylic acid cycle adsorbs free copper ions from the cytoplasmic matrix, leading to the aggregation of lipid acylated proteins, which contributes to cuproptosis. Furthermore, the co-protein levels of Fe-S clusters are reduced. Both of these induce a proteotoxic stress response and eventually lead to cell death (Tsvetkov et al., 2022). In addition, the copper levels are probably correlated with ferroptosis, which is caused via ROS accumulation (Gao et al., 2021). However, the exact molecular mechanism of cuproptosis has not yet been elucidated. This new cell death mechanism can help researchers improve their insights into the functioning of copper in cancer and provide potential ideas for the development of novel anti-tumor drug development.

Immune checkpoint blockade therapies have been used for a variety of cancers, most of which are CTLA-4 and PD-1/PD-L1 (Darvin et al., 2018). Several drugs have been developed for clinical applications, such as tremelimumab, ipilimumab, avelumab, atezolizumab, and pembrolizumab (Bagchi et al., 2021). However, not all patients respond positively to immunotherapy, and there are no predictors of benefit from immunotherapy (Bagchi et al., 2021). Therefore, uncovering the clinical indicators that have the potential to positively predict the response to immunotherapy will provide guidance for clinical practice and the implementation of precise and personalized treatment modalities for cancer patients, which is expected to improve a patient's quality of life as well as overall survival rates. The benefits of adjuvant chemotherapy in the adjuvant treatment of lymph node–positive colon cancer are well established, and standard treatment options include fluorouracil (FU) or capecitabine with or without oxaliplatin (Gelibter et al., 2019). As there are individual differences between patients and chemotherapy drugs that may induce drug resistance in tumor cells, predicting the therapeutic effect of chemotherapy has become the focus of clinical attention.

This study utilized TCGA and GEO databases of COAD expression profiles to identify cuproptosis-related lncRNAs (CRLs). The RiskScore for COAD prognosis was constructed using the WGCNA and COX regression. Furthermore, we investigated the potential link between RiskScore and prognosis, immune microenvironment, and treatment response. Our study potentially provides a novel approach for assessing COAD prognosis and treatment guidance that can be applied in a clinical setting.

## Materials and methods

### Data set downloading and pre-processing

RNA-Seq data, clinical data, and SNV data for the TCGA-COAD sequencing project were all from the TCGA portal (https://portal.gdc.cancer.gov/). Meanwhile, the GSE39582 and GSE17538 cohorts with the corresponding clinical information were all from the GEO portal (https://www.ncbi.nlm.nih.gov/geo/). A total of 13 cuproptosis-associated genes were taken from Tsvetkov et al. (2022). This study considered the TCGA-COAD cohort as the training set, and the GSE39582 and GSE17538 cohorts as the external validation set.

The RNA-Seq data of TCGA-COAD were processed in three steps: primary solid tumor samples with expression profiles and overall survival times were retained as the training subjects (Miller et al., 2020), Ensembl IDs were converted to Gene Symbols (Punt et al., 2017), and expression cases with multiple Gene Symbols were taken as their median (Ferlay et al., 2021).

The following processes were performed for the GSE39582 and GSE17538 data sets: samples with expression profiles and overall survival information were retained as training subjects (Miller et al., 2020), and probe sequences were re-annotated using the SeqMap to convert probes to Gene Symbol (Punt et al., 2017).

### Investigation of the correlation of COAD prognosis with cuproptosis score

Based on 13 cuproptosis-related genes (CRGs), the cuproptosis score for each sample in the TCGA-COAD and GSE39582 cohorts was calculated by the single-sample gene set enrichment analysis (ssGSEA) (Barbie et al., 2009). By means of the surv_cutpoint function integrated with the survminer package, the samples were classified according to the best cutoff parameters of survival information into different cuproptosis score groups for the K-M survival analysis.

### WGCNA analysis to identify cuproptosis-associated gene modules and lncRNAs

The WGCNA package (Langfelder and Horvath, 2008) was employed to build a weighted gene co-expression network on tumor samples in the TCGA-COAD cohort. The module eigengene (ME) of gene modules screened via the principal component analysis was calculated. The gene modules and lncRNAs that significantly correlated with cuproptosis via the Pearson correlation were screened to compare the correlation of ME with cuproptosis, age, and stage. The Kyoto Encyclopedia of Genes and Genomes (KEGG) biological pathways involved in gene modules were analyzed based on the WebGestaltR package (Liao et al., 2019).

### Identification of prognostic CRLs and construction of predictive model in COAD

The training and validation cohorts were divided into the TCGACOAD cohort according to the 7:3 ratio. Univariate COX regression analysis was considered on CRLs to dig out COAD prognosis–related lncRNAs (*p* < 0.05). The approach with LASSO COX was then conducted using the glmnet package (Simon et al., 2011) to select the best lambda to compress the number of lncRNAs to refine the model based on 10-fold cross-validation. Ultimately, the approach with multivariate COX was performed to construct the best clinical prognostic model via selecting the minimum Akaike information criterion (Pages et al., 2018) value using the stepAIC function of the MASS package (https://cran.r-project.org/web/packages/MASS/MASS.pdf). The RiskScore of the prognostic model was obtained via the following formula:

$$RiskScore=\sum {coef}_{i}*\mathrm{Exp}\;{lncRNAs}_{i}$$

where coef indicates the regression coefficient of lncRNAs_i_ and Exp lncRNAs_i_ indicates the expression of lncRNAs_i_.

### Predictive evaluation by RiskScore

High- and low-score groups of COAD patients in the training set were categorized by the best RiskScore grouping cutoff. The K-M analysis was applied to assess the overall survival (OS) differences between the different groups. The ROC curve was drawn using the timeROC package to obtain the area under ROC (AUC) values (Blanche et al., 2013). The model predictive ability was then evaluated in the validation cohort and external cohorts GSE39582 and GSE17538.

### Differences in clinicopathological characteristics and SNV in high- and low- score groups

We further explored the differences in clinicopathological characteristics in distinct subgroups in the TCGA-COAD, GSE39582, and GSE17538 cohorts using chi-square tests to assess categorical data. The OS differences in distinct clinicopathological characteristics subgroups were assessed by the K-M survival curve. Mutation data processed by Mutect2 were downloaded from the TCGA dataset. Genomic mutation differences between samples were resolved using the Fisher’s test for those with mutation frequencies greater than or equal to 3, and the mutation landscape Waterfall map was plotted using the GenVisR package (Skidmore et al., 2016). Finally, the immune landscape signature (fraction altered, the number of segments, tumor mutation burden, and homologous recombination defects) of COAD were downloaded from a previous study (Thorsson et al., 2018) using the Wilcoxon test for difference comparison of these immune signatures in different risk groups (*p* < 0.05).

### TME differences

In the GSE39582 cohort, immune cell infiltration in COAD patients was assessed by the expression of immune cell marker genes. Immune cell infiltration in TME was calculated by MCP-counter (Becht et al., 2016) and ssGSEA, respectively. StromalScore, ImmuneScore, and ESTIMATEScore were calculated using the Estimation of STromal and Immune cells in MAlignant Tumor tissues using Expression data (ESTIMATE) (Yoshihara et al., 2013). Then, the immune checkpoint expression (Danilova et al., 2019) was contrasted in different risk groups. A two-tailed *t*-test was performed to analyze the differences in StromalScore, ImmuneScore, ESTIMATEScore, and immune checkpoint expression levels. *p* < 0.05 was considered statistically different.

### Immunotherapy response/chemotherapy drug sensitivity analysis

The TIDE scores were sourced from the Tumor Immune Dysfunction and Exclusion database (TIDE, http://tide.dfci.harvard.edu/) of the GSE39582, GSE17538, and TCGA-COAD cohorts. Higher TIDE scores indicate higher vulnerability to immune escape, indicating less benefit from taking immunotherapy (Jiang et al., 2018). The half maximal inhibitory concentration (IC50) of COAD patients to the conventional chemotherapeutic agents, such as cisplatin, PHA-665752, AZ628, crizotinib, S-trityl-L-cysteine, and imatinib, was then analyzed using pRRophetic to assess chemotherapeutic drug sensitivity (Geeleher et al., 2014).

### Construction of decision trees and nomogram

Combining the clinicopathological characteristics information of the GSE39582 cohort, a decision tree was constructed using the rpart package (https://cran.r-project.org/web/packages/rpart/index.html) to identify the clinicopathological characteristics that affect prognosis. Univariate and multivariate COX analyses were conducted to identify independent clinical prognostic factors. A nomogram to predict 1-, 3-, and 5-year survival in patients with COAD was constructed via the rms package (https://cran.r-project.org/web/packages/rms/rms.pdf), and calibration curves reflected the predictive accuracy of the nomogram. Then, the ROC curve was plotted to evaluate the prognostic predictive power of the nomogram, RiskScore, TNM stage, and age. Finally, the clinical benefits of RiskScore and the nomogram were evaluated using the decision curve.

### Statistical analysis

The data analysis in this study was conducted with R 4.1.1. The *t*-test, chi-square test, Wilcoxon test, and Fisher’s test were applied for statistical analysis of the data. The K-M method was used for the survival analysis of groups, and the log-rank test was used to assess the significance of differences. In this study, *p* < 0.05 was considered statistically significant. The Sangerbox contributed to this report (Shen et al., 2022).

## Results

### High cuproptosis score reduced OS in COAD

**FIGURE 1:**
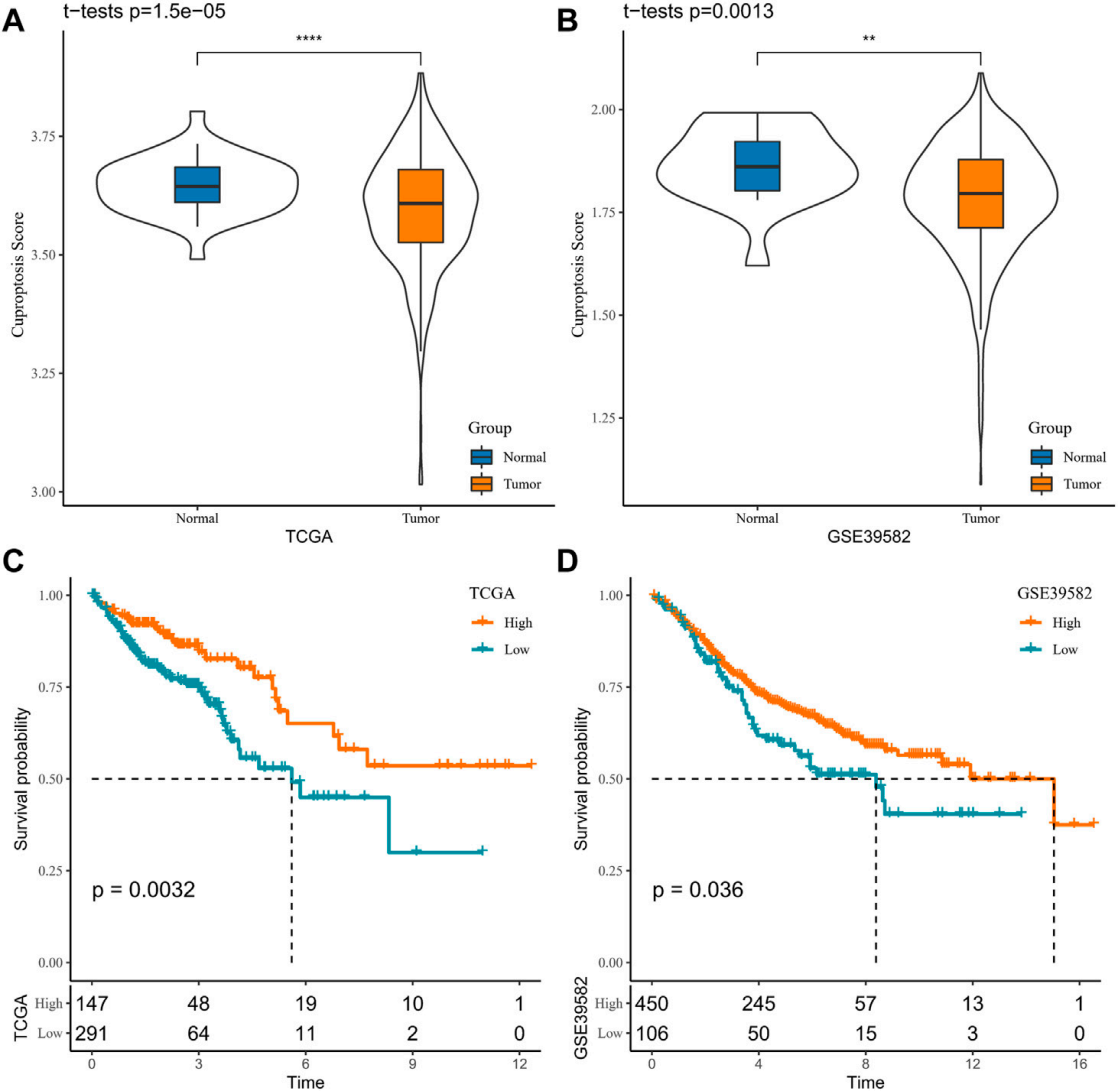
Association between cuproptosis and COAD prognosis in TCGA-COAD and GSE39582 cohorts **(A, B)**. Sample cuproptosis score in tumor group and normal group **(C, D)**. K-M survival curves for high- and low-cuproptosis-score groups.

Initially, the cuproptosis score of each patient was assessed utilizing ssGSEA in the TCGA-COAD and GSE39582 cohorts. When compared with normal samples, it was visualized that the cuproptosis score of the tumor tissue was significantly higher (*p* = 1.5e-05/0.0013, Figures 1A, B). Then, based on the best cutoff values, the samples in the TCGA-COAD and GSE39582 cohorts were clustered into high- and low-cuproptosis-score groups. To explore the potential relationship between the cuproptosis score and OS, we plotted K-M survival curves. The OS of patients with low scores in the TCGA-COAD and GSE39582 cohorts was significantly better (*p* = 0.0032/0.036, Figures 1C, D). These results imply an association between the cuproptosis score obtained based on CRGs and COAD prognosis.

### Identification of cuproptosis-related gene modules and lncRNAs

**FIGURE 2:**
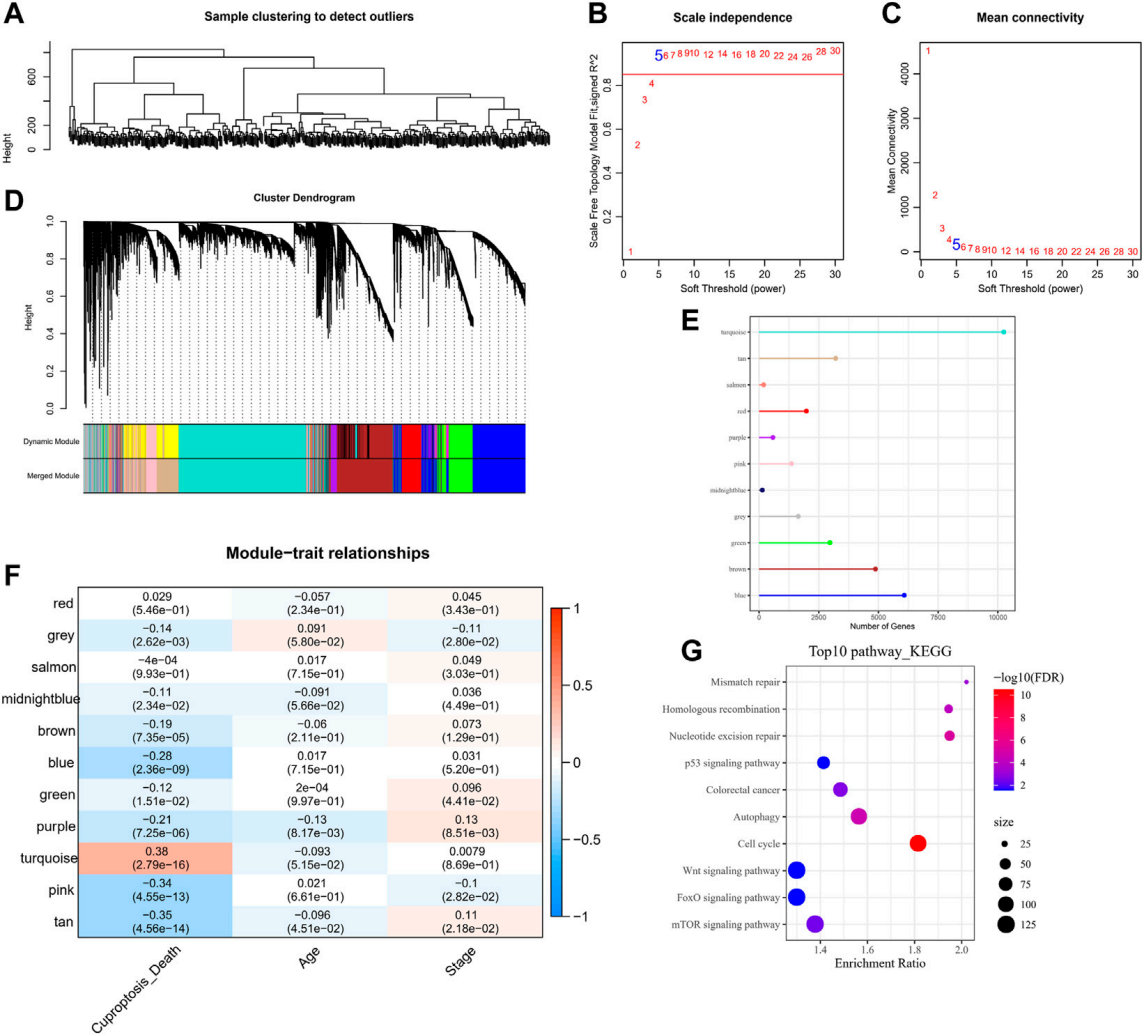
WGCNA analysis on tumor samples in the TCGA-COAD cohort. **(A)** Hierarchical clustering tree of tumor samples. **(B)** Analysis of the scale-free fit index for various soft-thresholding powers (*β*). **(C)** Analysis of the mean connectivity for various soft-thresholding powers. **(D)** Dendrogram of all co-expressed genes/lncRNAs clusters based on a dissimilarity measure (1-TOM). **(E)** Number of genes within the 11-gene module. **(F)** Pearson correlation of 11-gene-module ME with clinical information. **(G)** KEGG pathway annotation of genes within the turquoise module.

The CRGs modules and lncRNAs were identified by the WGCNA. Five was determined as the optimal soft threshold in building a scale-free network, and 11 gene modules were obtained according to the average linkage hierarchical clustering and dynamic shearing tree (Figures 2A–D). The gene number of each module is shown in Figure 2E. It is observable that the turquoise module is noticeably positively related to cuproptosis (R = 0.38, *p* < 0.001), and the rest of the gene modules are negatively correlated with cuproptosis (Figure 2F). Therefore, we selected the turquoise module for the next analysis. Additionally, the KEGG enrichment analysis demonstrated that in the turquoise module, genes were mainly enriched in the p53 signaling pathway, colorectal cancer, mismatch repair, autophagy, and homologous recombination pathways (Figure 2G).

### Construction of a prognostic model related to cuproptosis in COAD

Using 1,897 CRLs identified in the WGCNA, prognosis-related lncRNAs in COAD were identified via univariate COX regression in the training set. A total of 78 lncRNAs capable of influencing the OS of COAD were identified. Owing to the high number, which was not conducive to clinical detection, the LASSO COX compressed model was implemented. For this procedure, the model was found to be optimal at lambda = 0.04559461 under 10-fold cross-validation, which contained RFPL3S, AC034236.2, EIF3J-DT, ZEB1-AS1, AL731533.2, LINC00638, AC008494.3, and AC016027.1. Finally, multivariate COX regression identified five lncRNAs associated with CRGs (Supplementary Figures S1A–C) to construct a clinical prediction model for COAD prognosis (Supplementary Figures S1D–F). The patient’s RiskScore was computed by using the formula: RiskScore = −1.177 × AC008494.3 + 1.098 × EIF3J-DT − 0.981 × AC016027.1 + 0.813 × AL731533.2 + 0.651 × ZEB1_AS1.

**FIGURE 3:**
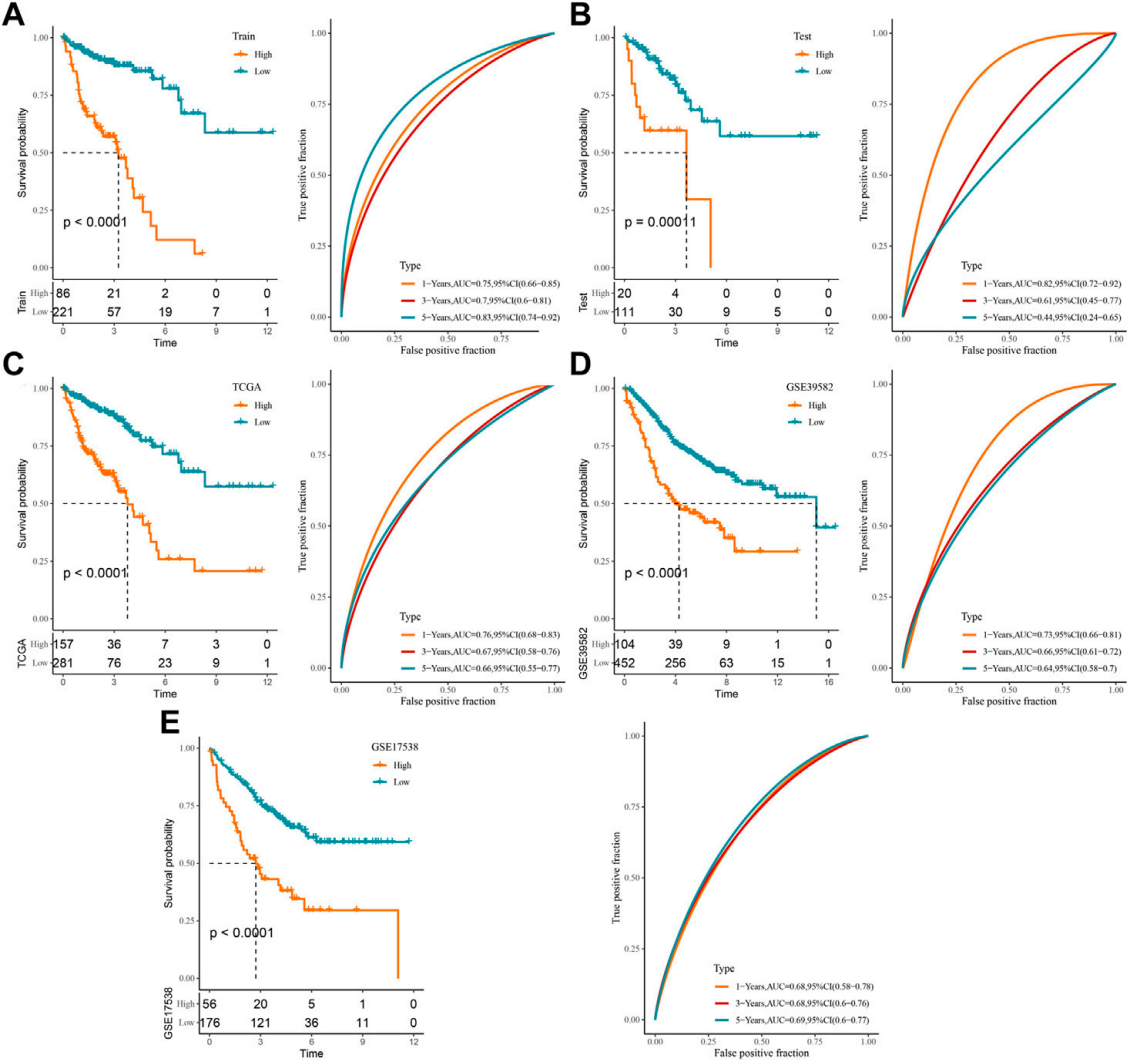
Construction of a clinical prognostic risk model for COAD: **(A)** training set, **(B)** validation set, **(C)** TCGA-COAD, **(D)** GSE39582, **(E)** GSE17538 K-M survival curves and ROC curves in the high-score and low-score groups.

Based on this formula and the optimal group cutoff determined by the survminer package, the COAD samples in the training and validation sets were divided into high-score and low-score groups, and we plotted the K-M survival curves and ROC curves for prognostic assessment. It was observed that patients in the high-score group in the training set had worse OS (*p* < 0.0001), and the ROC curve showed that the AUC values of RiskScore for predicting 1-, 3-, and 5-year survivals were 0.75, 0.7, and 0.83, respectively (Figure 3A). The high-score group in the validation set had a better prognosis (*p* = 0.00011), with AUC values of 0.82, 0.61, and 0.44 at 1, 3, and 5 years, respectively (Figure 3B). It could be seen that the AUC values of RiskScore in the validation set for predicting 3-year and 5-year survival of patients were not satisfactory, which may be attributed to the small number of samples. Therefore, the model was verified in the TCGA-COAD, GSE39582, and GSE17538 cohorts, respectively. The TCGA-COAD, GSE39582, and GSE17538 cohorts all exhibited worse prognosis in the high-score group, and RiskScore predicted 1-, 3-, and 5-year AUC values greater than 0.6 with a sufficiently sizeable sample (Figures 3C–E). These results illustrate that RiskScore exhibited good overall predictive performance.

### Differences in clinicopathological characteristics and mutational characteristics among high- and low-score groups

**FIGURE 4:**
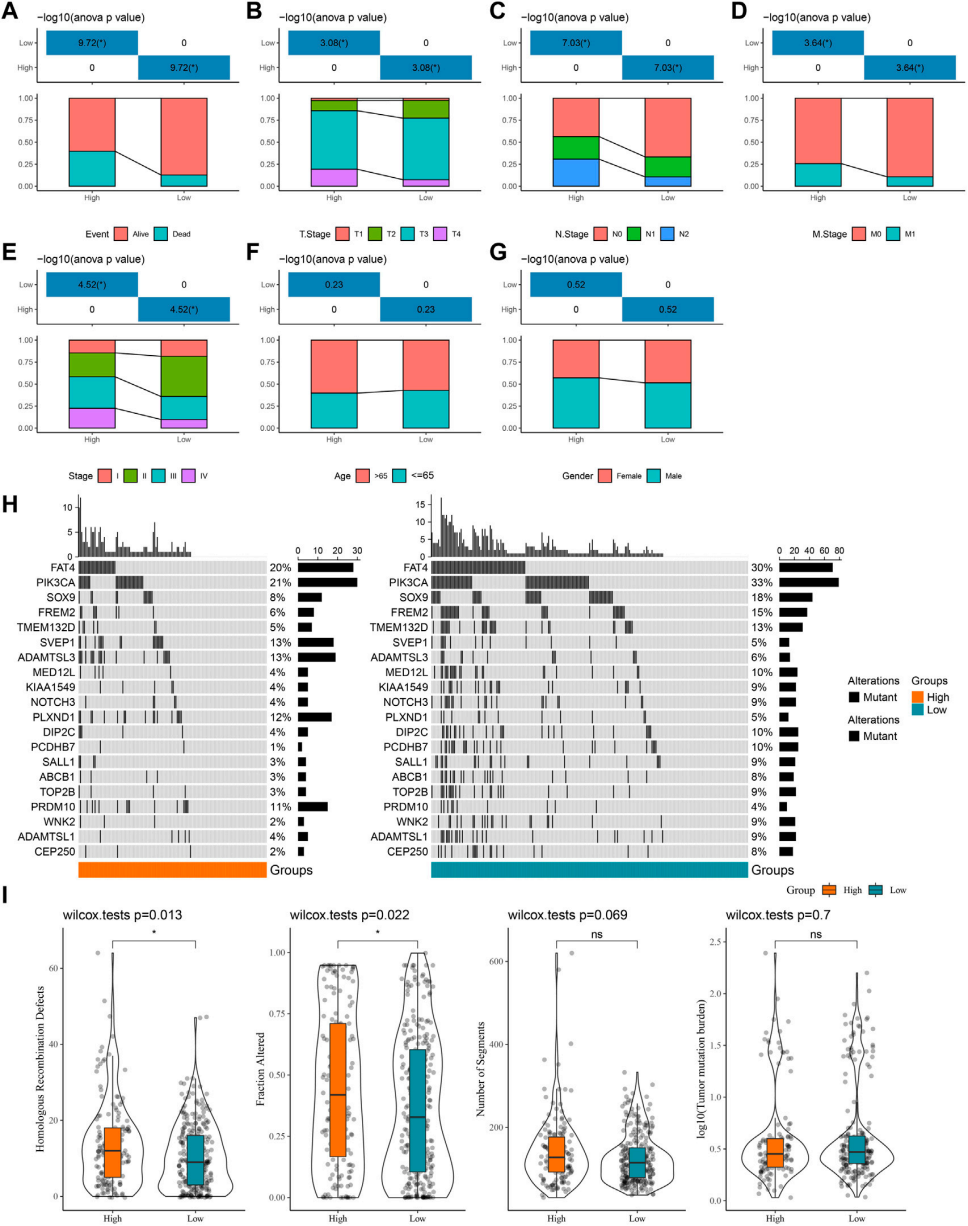
Differences in clinicopathological characteristics as well as mutational characteristics among high- and low-score groups. **(A–G)** Statistics of event, T stage, N stage, M stage, stage, age, and gender information of patients in the TCGA-COAD cohort in the high-score and low-score groups, with the lower half being the proportion of cases and the upper half being statistically significant statistics. **(H)** Waterfall plot of the top 20 genes in mutation frequency. **(I)** Boxplots of homologous recombination defects, fraction altered, number of segments, and tumor mutation burden.

Furthermore, we counted the differences in clinicopathological characteristics between the high- and low-score groups in the TCGA-COAD, GSE39582, and GSE17538 cohorts. In the TCGA-COAD cohort, we found a higher proportion of patient deaths, high tumor TNM stage, and Stage patients in the high-score groups (*p* < 0.05) (Figures 4A–E), whereas there were no significant differences in age and gender (Figures 4F, G). In the GSE39582 cohort, the event, TNM stage, and stage trends were consistent with those of the TCGA-COAD cohort, with the difference that there were more elderly patients in the high-score group (Supplementary Figures S2A–G). By contrast, a higher proportion of deaths and high-stage conditions were observed in the high-score groups in GSE17538 (Supplementary Figures S3A–E). These results have further confirmed that patients in the high-score group have a poorer prognosis and that the majority of the patients have a higher clinicopathological stage. Naturally, genomic mutations were counted in the TCGA-COAD cohort for each sample in the high- and low-score groups and waterfall plots were drawn to show the 20 genes with the highest mutation frequencies (Figure 4H). We found that the majority of the 20-gene mutations were higher in the low-score group, which may be one of the factors for a good prognosis of the low-score group. Finally, we downloaded the cancer immune landscape of COAD from previous studies and found that homologous recombination defects and fraction altered scores were higher in the high-score group (*p* < 0.05), while the number of segments and tumor mutation burden were not significantly different (Figure 4I).

### Performance of RiskScore in clinicopathology subgroups

**FIGURE 5:**
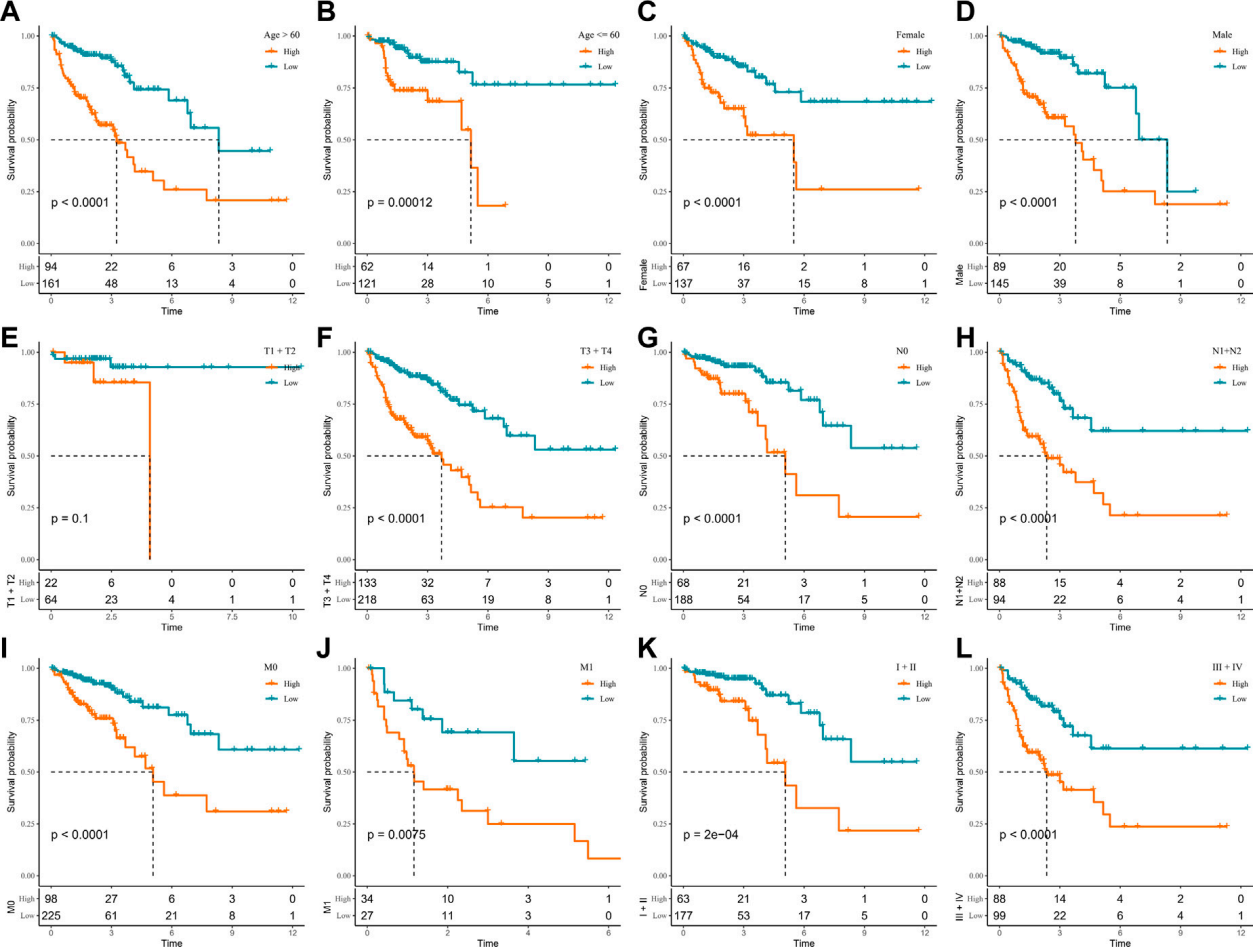
K-M survival curves for high- and low-score groups in the clinicopathological subgroups of the TCGA-COAD cohort. **(A, B)** Age subgroup, **(C, D)** gender subgroup, **(E, F)** T stage subgroup, **(G, H)** N stage subgroup, **(I, J)** M stage subgroup, and **(K, L)** stage subgroup.

**FIGURE 6:**
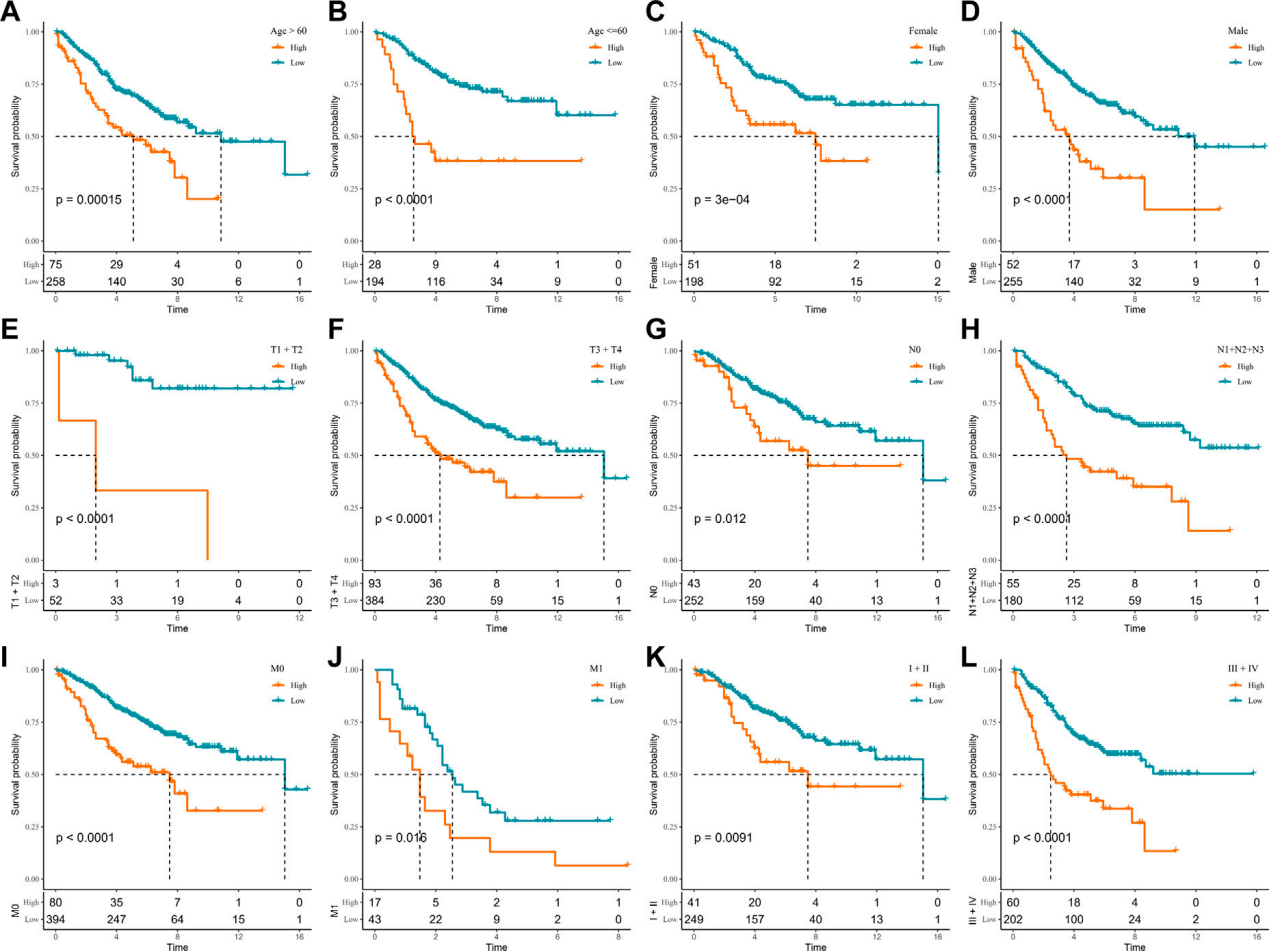
K-M survival curves for high- and low-score groups in the clinicopathological subgroups of the GSE39582 cohort. **(A, B)** Age subgroup, **(C, D)** gender subgroup, **(E, F)** T stage subgroup, **(G, H)** N stage subgroup, **(I, J)** M stage subgroup, and **(K, L)** stage subgroup.

In the TCGA-COAD cohort, to demonstrate that RiskScore is an equally favorable predictor in different clinical characteristic subgroups, we sketched the K-M curves in different subgroups of the high- and low-score groups. We noted that among the subgroups of age, gender, TNM stage, and stage, all subgroups showed poor prognosis in the high-score group except for the T1 + T2 subgroups (Figures 5A–L). The following analyses were conducted in the GSE17538 and GSE39582 cohorts. In addition, a similar analysis was done in the GSE17538 and GSE39582 cohorts. The outcomes of the overall survival showed that the clinicopathology of all patients with a high score was significantly shorter (Figures 6A–L, Supplementary Figures S4A–I). The aforementioned results suggest that RiskScore is a reliable prognostic indicator.

### Association between RiskScore and immune microenvironment

**FIGURE 7:**
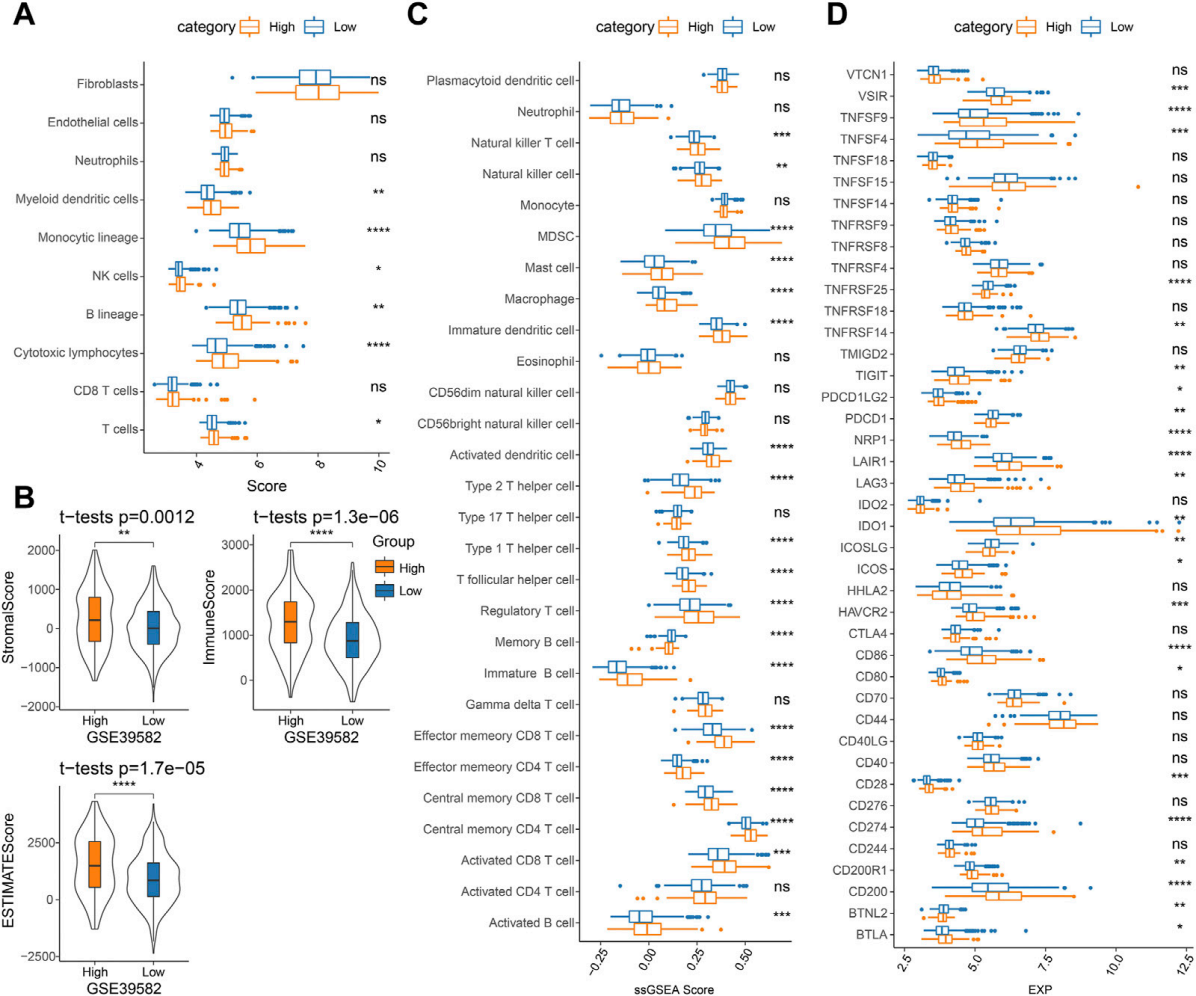
Analysis of immune cell infiltration in TME in the GSE39582 cohort: **(A)** MCP-counter analysis, **(B)** ESTIMATE analysis, **(C)** ssGSEA analysis, and **(D)** immune checkpoint gene expression.

To determine differences in the abundance of infiltrating immune cells in the TME of COAD patients in the high- and low-score groups, MCP-counter, ssGSEA, and ESTIMATE algorithms were employed to analyze the differences in the immune microenvironment of GSE39582 cohort patients. Figure 7A displays the results of the MCP-counter analysis. Significant differences could be observed in the infiltration of NK cells, myeloid dendritic cells, T cells, monocytic lineage, cytotoxic lymphocytes, and B lineage in different risk groups. Then, the stromal and immune cell scores in the TME were analyzed via the ESTIMATE. The results showed significantly higher StromalScore, ImmuneScore, and ESTIMATEScore in the high-score group (Figure 7B). Similarly, ssGSEA analysis detected most of the higher immune cell infiltration scores in the high-score group (Figure 7C). Additionally, in the high- and low-score groups, a comparison of the immune checkpoint gene expression showed a high expression of the vast majority of immune checkpoint genes in the high-score group (Figure 7D). The TCGA-COAD and GSE17538 cohorts were similarly processed and showed similar trends to those in the GSE39582 cohort (Supplementary Figures S5A–D, Supplementary Figures S6A–D).

### Association between RiskScore and immunotherapy response/chemotherapy sensitivity

**FIGURE 8:**
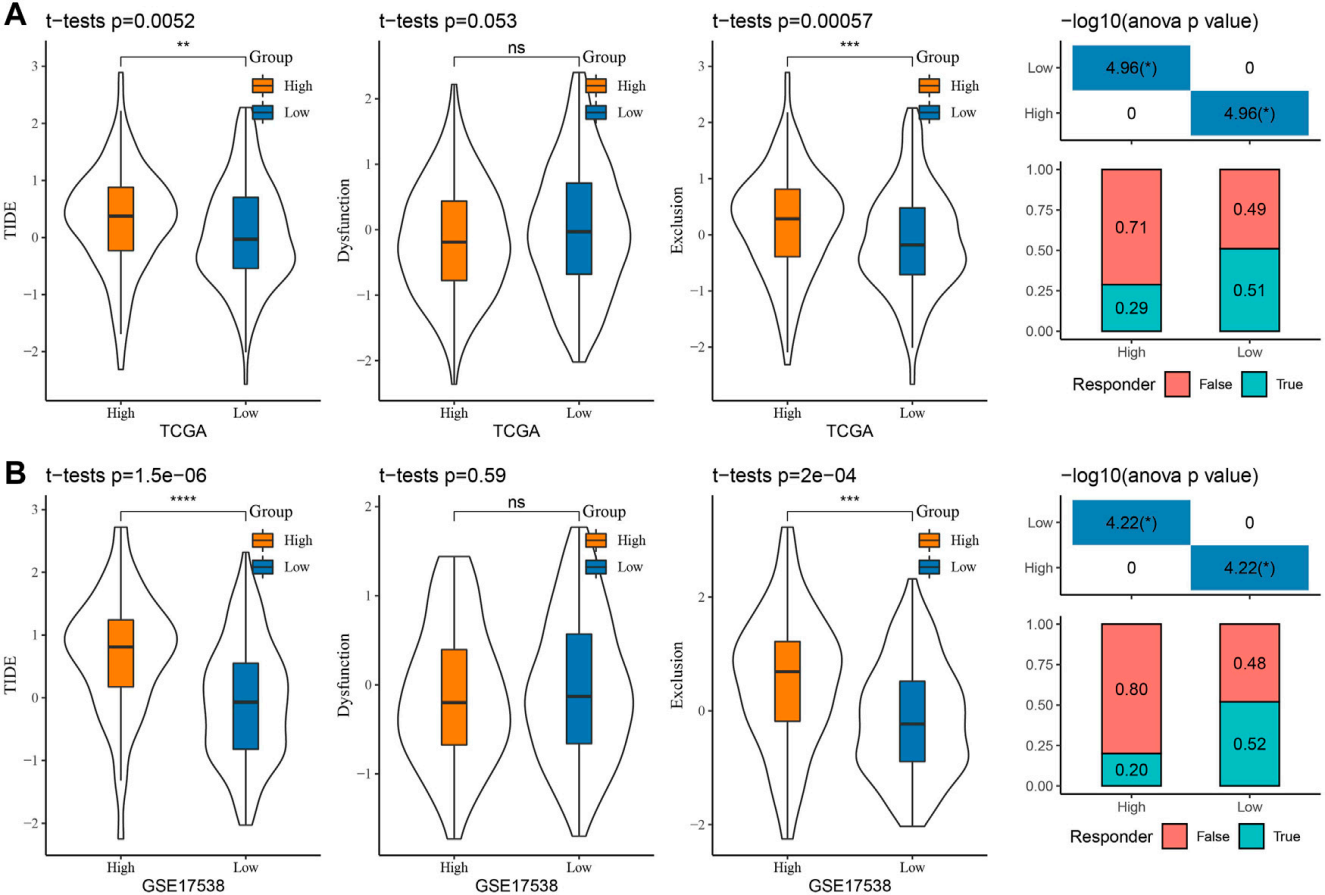
Prediction of response to immunotherapy: **(A)** TCGA-COAD and **(B)** GSE17538 TIDE score and prediction of response to immunotherapy.

Presently, most cancer patients do not respond well to immunotherapy (Bagchi et al., 2021). To clarify the potential of RiskScore in predicting immunotherapy response in COAD patients, we analyzed the variability in immunotherapy response in two risk score groups. We calculated the TIDE score, dysfunction score, and exclusion score in the TIDE software for COAD patients in the TCGA-COAD and GSE17538 cohorts. We observed higher TIDE scores in the high-score group in the two cohorts. The lower dysfunction scores and higher exclusion scores were observed in both TCGA-COAD and GSE17538 in the high-score group (Figures 8A, B). These overall results have indicated that patients with a low RiskScore possibly responded better to immunotherapy, while an increased potential for immune escape occurred in the high-score group, resulting in poor immunotherapy outcomes. Meanwhile, to predict the response of RiskScore to immunotherapy, we found that the proportion of patients in the low-score group responding to immunotherapy was 51% and 52% in the TCGA-COAD and GSE17538 cohorts, respectively (Figures 8A, B).

**FIGURE 9:**
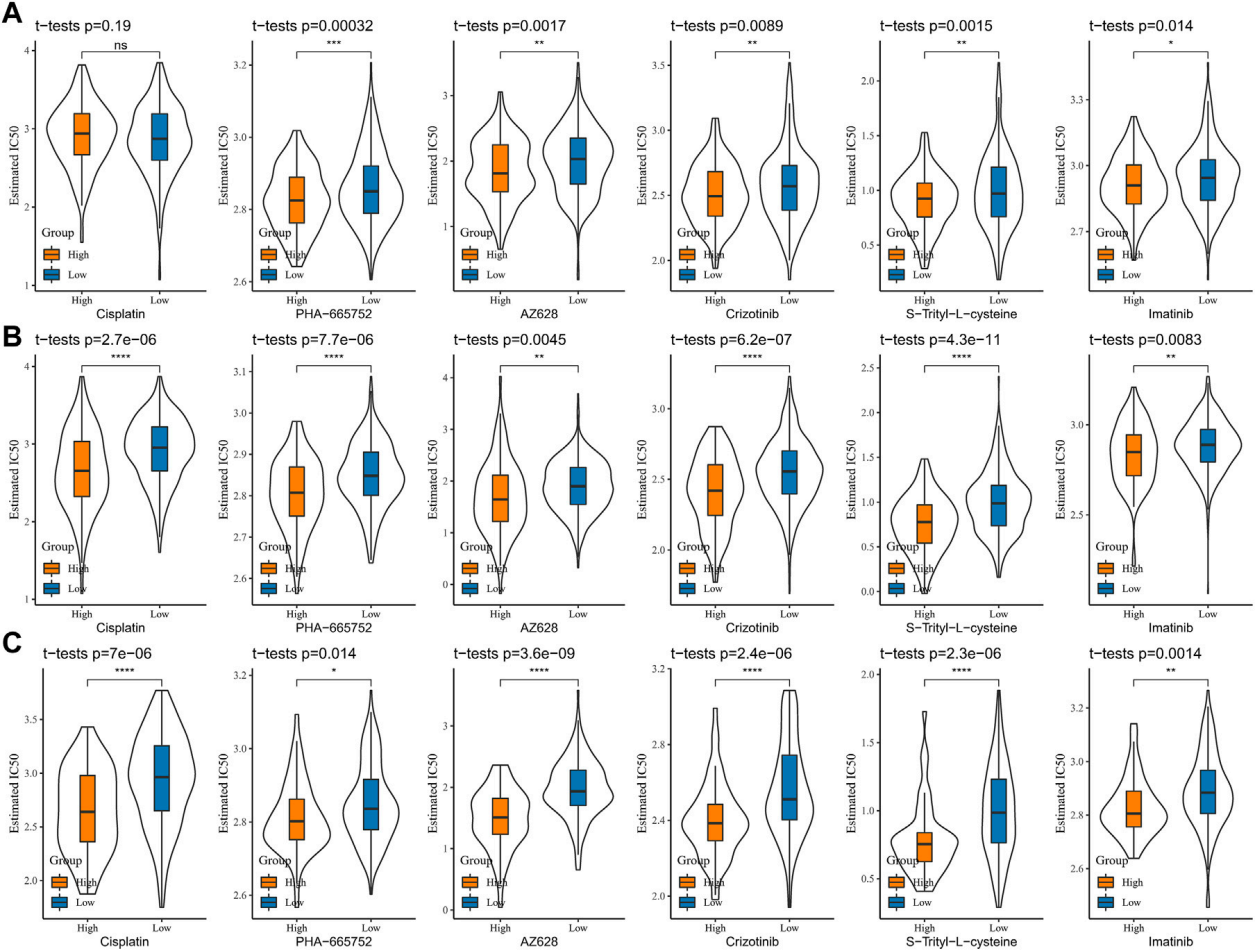
Sensitive analysis of chemotherapy drugs **(A)** TCGA-COAD, **(B)** GSE39582, and **(C)** GSE17538, and the IC50 of cisplatin, PHA-665752, AZ628, crizotinib, S-trityl-L-cysteine, and imatinib.

To analyze the sensitivity of COAD patients to conventional chemotherapeutic drug treatment, the IC50 of cisplatin, PHA-665752, AZ628, crizotinib, S-trityl-L-cysteine, and imatinib was assessed via the pRRophetic package. The results in the TCGA-COAD, GSE39582, and GSE17538 cohorts were consistent, with significantly higher IC50s for all six drugs in the low-score group than in the high-score group, indicating that patients in the low-score group were more sensitive to cisplatin, PHA-665752, AZ628, crizotinib, S-trityl-L-cysteine, and imatinib treatments (Figures 9A–C). The abovementioned results indicate that low-score COAD patients respond better to immunotherapy and chemotherapy and that RiskScore is a potential biomarker for predicting treatment response.

### Nomogram for predicting COAD survival

**FIGURE 10:**
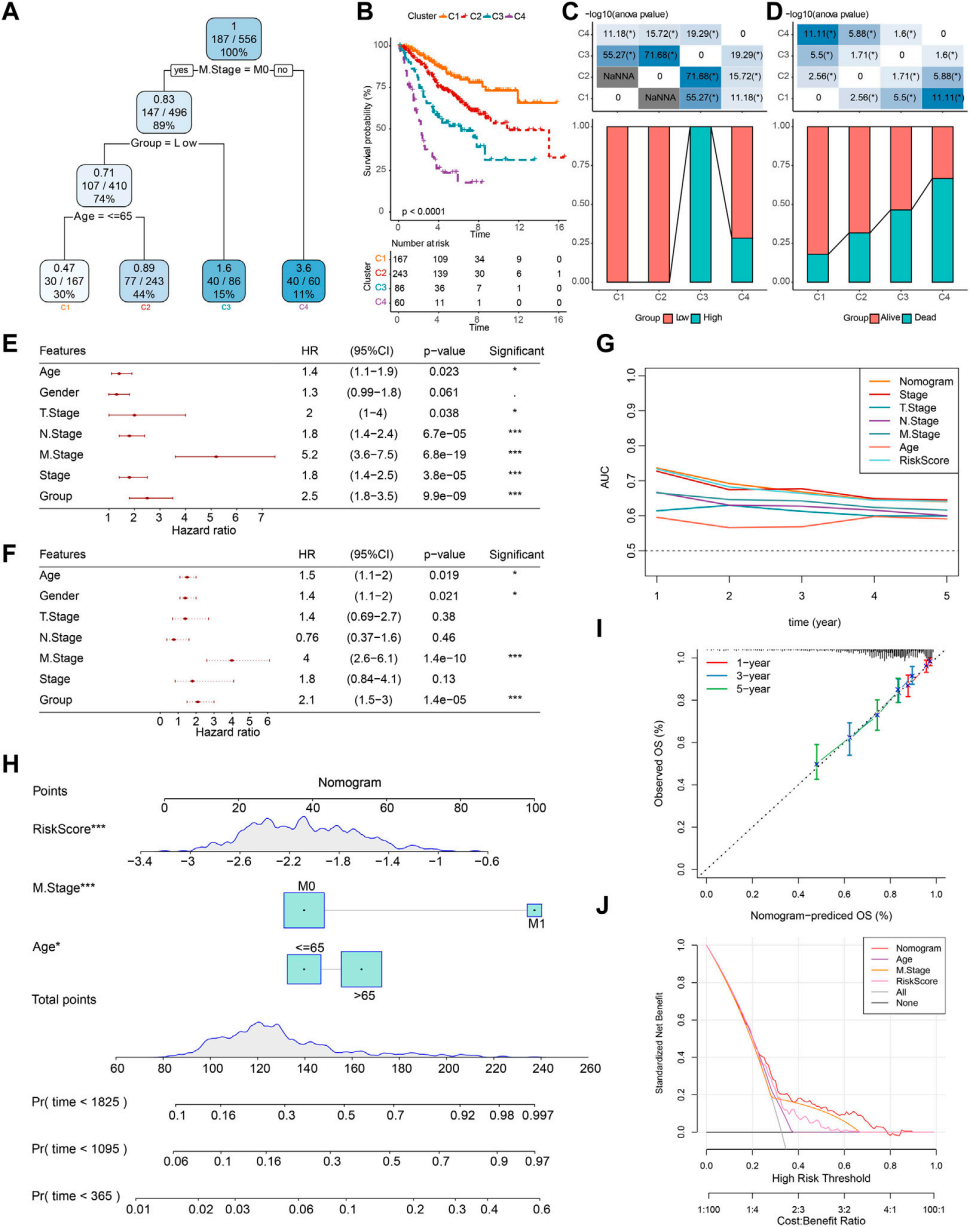
Construction of decision trees and nomogram. **(A)** Patients with full-scale annotations that include RiskScore, stage, gender, and age were used to build a survival decision tree to optimize risk stratification. **(B)** Significant differences of K-M survival were observed among the four risk subgroups. **(C, D)** Distribution of patients in different subgroups. **(E, F)** Univariate and multivariate COX analysis of RiskScore and clinicopathological characteristics. **(G)** Compared with other clinicopathological features, the nomogram exhibited the most powerful capacity for survival prediction. **(H)** Nomogram predicting survival at 1, 3, and 5 years for patients with COAD. **(I)** Calibration curves for 1-, 3-, and 5-year survival rates. **(J)** Decision curves for nomogram.

Based on age, gender, TNM stage, stage, and group, a decision tree was constructed, and four subgroups, namely, C1, C2, C3, and C4 were determined (Figure 10A). The decision tree showed that group, age, and M stage were the major contributors in the subgroups. The K-M survival analysis showed significant differences in OS among the four subgroups, with the best prognosis in the C1 subgroup and the worst prognosis in the C4 subgroup (Figure 10B). We found that all patients in the C1 and C2 subgroups were in the low-score group, and all patients in the C3 subgroup were in the high-score group (Figure 10C). The differences in the survival status of patients in different subgroups are shown in Figure 10D. Univariate and multivariate COX regression analyses were performed based on age, gender, TNM stage, stage, and group to validate whether the group was an independent prognostic factor for COAD. The results showed that the group was an independent prognostic factor for COAD (*p* < 0.001) (Figures 10E, F). The ROC curves showed that there was a positive predictive power with stage, TNM stage, age, and RiskScore (Figure 10G).

Then, based on the major contributors in the decision tree, a nomogram was built to predict the 1-, 3-, and 5-year OS of COAD patients (Figure 10H). The calibration curves showed that the prediction curves for predicting the 1-, 3-, and 5-year OS of COAD based on the nomogram fit well with the actual observed curves, which suggests that the nomogram had a promising predictive performance (Figure 10I). Finally, the DCA was used to assess model reliability, and it was observed that both the RiskScore and nomogram achieved significantly higher benefits than the extreme curves, and both the nomogram and RiskScore showed the strongest survival prediction ability as shown in Figures 10G, J when compared to other clinicopathological characteristics.

## Discussion

In recent years, despite early screening measures such as colonoscopy and tumor biomarkers testing, the incidence of COAD had been greatly reduced (Sung et al., 2021). However, frustratingly, features such as metastasis, recurrence, and chemotherapy resistance in COAD patients have caused the mortality rate to remain high, with a 5-year survival rate of only 12% according to statistics (Shah et al., 2016; Miller et al., 2019; Parseghian et al., 2019). The review by Chen and Shen (2020) has indicated that lncRNAs play a crucial role in the pathogenesis, progression, and treatment of COAD and that lncRNAs may be potentially promising biomarkers for tumor therapy. The report by Deng et al. (2017) has indicated that serum lncRNAs are a novel detection and prognostic biomarker that are easily accessible, non-invasive, and appropriately tested at a cost relative to conventional colonoscopy, and notably, lncRNAs demonstrate excellent accuracy and utility in clinical diagnosis and prognostic predictive management. Several COAD prognosis–related lncRNAs have been reported. lncRNA LBX2-AS1 contribute to the development of distal metastasis in COAD patients by regulating the miR-627-5p/RAC1/PI3K/AKT axis, and LBX2-AS1 is a novel biomarker of prognosis in COAD (Fang et al., 2022). O'Brien et al. (2022) noted that elevated lncRNA H19 expression affects the KRAS mutation status and increases the risk of distal metastasis in COAD patients, and H19 could be a potential molecular biomarker for the prognosis and treatment of COAD. Cuproptosis is a newly discovered mode of cell death, and given the role of copper homeostasis in cancer progression and copper complexes as potential therapeutic options (Tsvetkov et al., 2022; Li, 2020; da Silva et al., 2022), this report is the first to screen prognostic CRLs in COAD via a bioinformatics approach.

First, we found an association between the cuproptosis score and COAD prognosis; based on this finding, the prognostic CRLs in COAD were obtained by the WGCNA and COX regression analysis, and the model contained AC008494.3, EIF3J-DT, AC016027.1, AL731533.2, and ZEB1-AS1. Previous studies have confirmed that AC008494.3, EIF3J-DT, AC016027.1, and ZEB1-AS1 are correlated with COAD prognosis, treatment resistance, and malignant disease progression. For example, two recent studies have reported that AC008494.3 and AC016027.1 are prognostic biomarkers for COAD and CRC, respectively (Wang et al., 2021; Zhang et al., 2021). EIF3J-DT was shown to be a prognostic biomarker associated with autophagy in COAD (Zhou et al., 2020). Through *in vivo* and *in vitro* assays, Luo et al. (2021) identified that lncRNA EIF3J-DT regulated autophagy and drug resistance in gastric cancer cells by targeting ATG14. lncRNA ZEB1-AS1 high expression was correlated with poor prognosis in COAD, and ZEB1-AS1 acted as a sponge in adsorbing to miR-455-3p and bound to it to regulate COAD cell growth and metastasis by targeting the action of PAK2 (Ni et al., 2020). While AL731533.2 is a newly identified COAD prognostic gene, its function remains to be further explored in subsequent studies. In this study, AC008494.3, EIF3J-DT, AC016027.1, AL731533.2, and ZEB1-AS1 were identified as COAD prognostic CRGs, of which AC008494.3 and AC016027.1 serve as protective factors, and EIF3J_DT, AL731533.2, and ZEB1_ AS1 are the as risk factors. Combined with previous studies, this study further demonstrates that these lncRNAs could be used as prognostic biomarkers for COAD, which might be complementary to existing prognostic biomarkers and have important implications for clinical guidance of COAD prognosis.

In the study, we observed differences between infiltrating immune cells and immunotherapy responses in patients with different RiskScore groups. Therefore, we speculate that this novel pattern of cell death in cuproptosis might have immunological relevance. AC008494.3 was reported to be the prognostic lncRNA associated with iron death in COAD (Xu et al., 2022). Interestingly, the accumulation of ROS caused by cuproptosis led to the ferroptosis of cells (Gao et al., 2021). A report indicated that ferroptosis and immune cells exhibited synergistic effects together to maintain tumor immune microenvironment homeostasis and that the process of ferroptosis in tumor cells could expose tumor antigens and enhance immunogenicity to enhance immunotherapeutic response (Zhang et al., 2019). The connection between cuproptosis, ferroptosis, and immunity indicates that CRGs are promising and potential therapeutic targets for future tumor therapy, and it is important to explore their mechanisms to guide anti-tumor therapy.

We observed lower immune checkpoint expression and TIDE scores in the low-score group, suggesting that low-score group patients were more sensitive to immunotherapy. It was also demonstrated that the percentage of patients responding to immunotherapy was higher in the low-score group. These results have suggested that RiskScore could be used as a novel biomarker for immunotherapy and immune response rates. ImmuneScore was confirmed to be a reliable prognostic indicator as a complement to TNM staging for predicting disease recurrence and mortality in COAD (Pages et al., 2018). The K-M curves, ROC curves, and nomogram all show RiskScore to be a reliable prognostic factor for COAD, and DCA curves show that both nomogram and RiskScore exhibit the most powerful survival prediction ability. These results show that RiskScore constructed from CRLs could be used as a complement to existing clinical prognostic factors.

Furthermore, in somatic mutation analysis, we found that the majority of patients in the low-risk group had a higher mutation frequency than patients in the high-risk group. It is generally believed that cancer cells with high mutation frequency can attenuate the immune checkpoint suppressive effect of immune cells on tumor sources (Schumacher and Schreiber, 2015; Miao et al., 2018). This observation is also supported by numerous studies. The adult neural tube cell tumors represent low tumor mutational burden (TMB) tumors that upregulated IDO1 expression in an inflammatory environment and enhanced IDO1 expression shaped a suppressive microenvironment by suppressing T-cell activity, which is one of the mechanisms of immune evasion in adult neural tube cell tumors (Folgiero et al., 2016; Munn and Mellor, 2016). Samstein et al. (2019) used the statistical analysis of 7,033 cancer patients (immunotherapy treatment: 1,662; no immunotherapy treatment: 5,371) with TMB data and survival time and found that high TMB improved survival in multiple cancers. Our study suggests that high-frequency somatic mutations in patients in the low-risk group, where cancer cells might be more easily recognized by immune cells and immune escape suppressed, could also have contributed to the good clinical outcome of patients.

We developed a novel and highly accurate cuproptosis-related prognostic factor for COAD patients. In addition, based on RiskScore and clinical factors, we developed a nomogram for predicting 1-, 3-, and 5-year OS in COAD. However, this study still has shortcomings. First, due to insufficient annotated files in the GEO database, this study was performed by dividing the TCGA cohort sample using a 7:3 ratio for model construction, and a large sample of data sets to construct the model would have been more convincing. Secondly, five CRLs were identified in this study, and the specific molecular mechanisms of action of these lncRNAs remain unclear, subsequent wet assays, such as Western blot, RT-qPCR, and immunohistochemistry need to be undertaken in further studies. These are important directions that will be explored in our subsequent work.

## Conclusion

We constructed a novel prognostic model using CRLs in COAD, and the CRLs in the model were potential therapeutic targets for COAD. According to our study, RiskScore is a new potential predictor of independent prognostic factors, immunotherapy response, and chemotherapy sensitivity in COAD. Based on the decision curves and nomogram, it was shown that RiskScore possesses strong robustness in prognostic assessment and is a reliable clinical prognostic guideline, providing a new scientific basis for the prognostic management of COAD.

## Data Availability

The original contributions presented in the study are included in the article/Supplementary Material; further inquiries can be directed to the corresponding authors.

## Abbreviations

AIC, Akaike information criterion; AUC, area under ROC curve; CDF, cumulative distribution function; COAD, colon adenocarcinoma; CRLs, cuproptosis-related lncRNAs; CRGs, cuproptosis-related genes; CC, colon cancer; ESTIMATE, Estimation of STromal and Immune cells in MAlignant Tumor tissues using Expression data; FC, fold change; FDR, false discovery rate; GEO, Gene Expression Omnibus; HR, hazard ratio; K-M, Kaplan–Meier; KEGG, Kyoto Encyclopedia of Genes and Genomes; LASSO, least absolute shrinkage and selection operator; ME, module eigengene; OS, overall survival; ROS, reactive oxygen species; ROC, receiver operating characteristic analysis; ssGSEA, single-sample gene set enrichment analysis; TCGA, The Cancer Genome Atlas; TIDE, Tumor Immune Dysfunction and Exclusion; TME, tumor microenvironment; WGCNA, weighted gene co-expression network analysis.
