# Supplementary figures and images for: Based on cuproptosis-related lncRNAs, a novel prognostic signature for colon adenocarcinoma prognosis, immunotherapy, and chemotherapy response
Source: Front Pharmacol. 2023 Jun 12;14:1200054. doi: 10.3389/fphar.2023.1200054 (PMC10291194; doi:10.3389/fphar.2023.1200054)

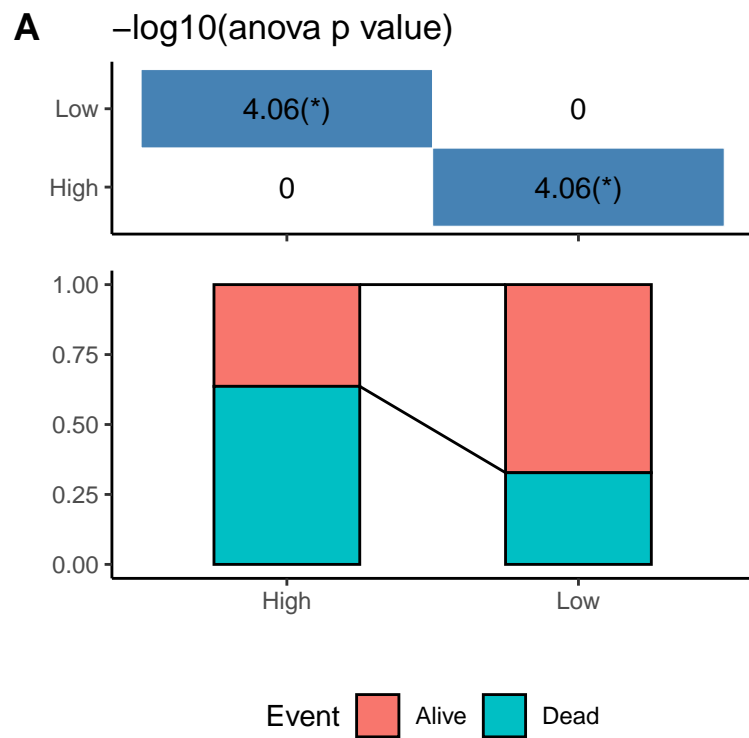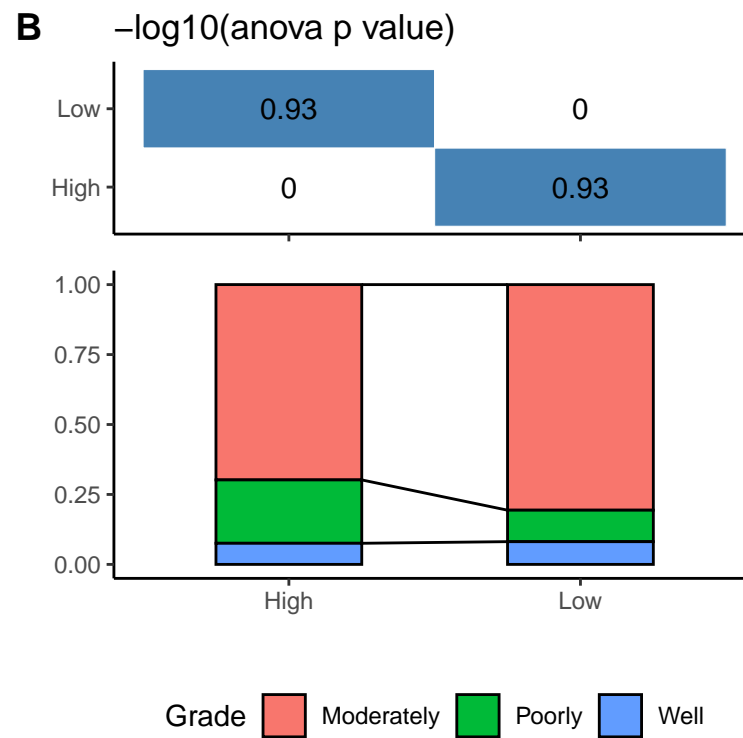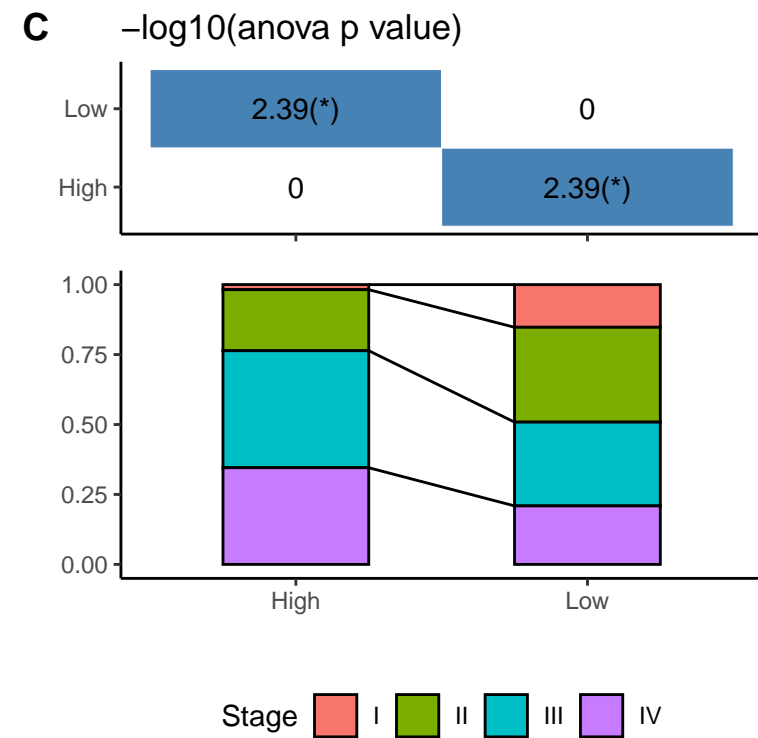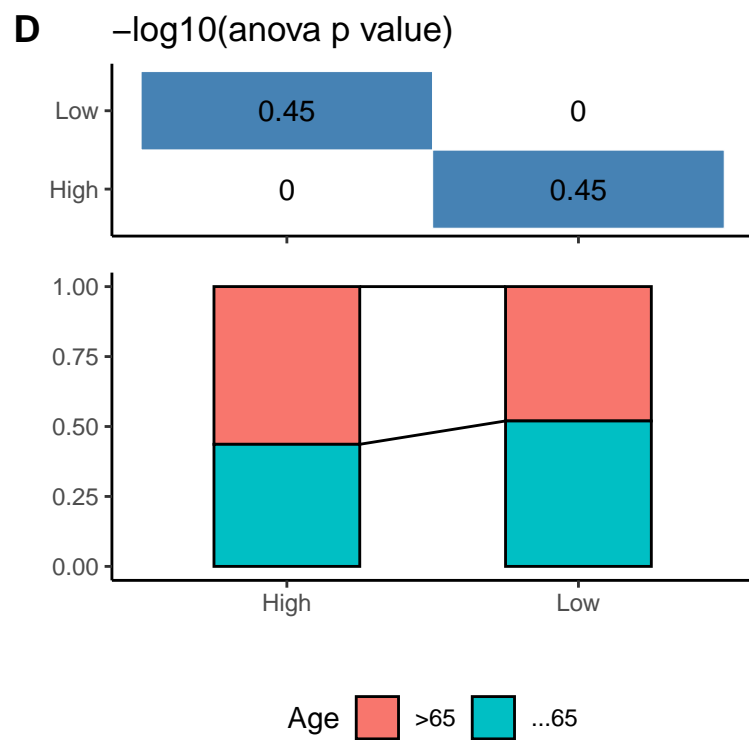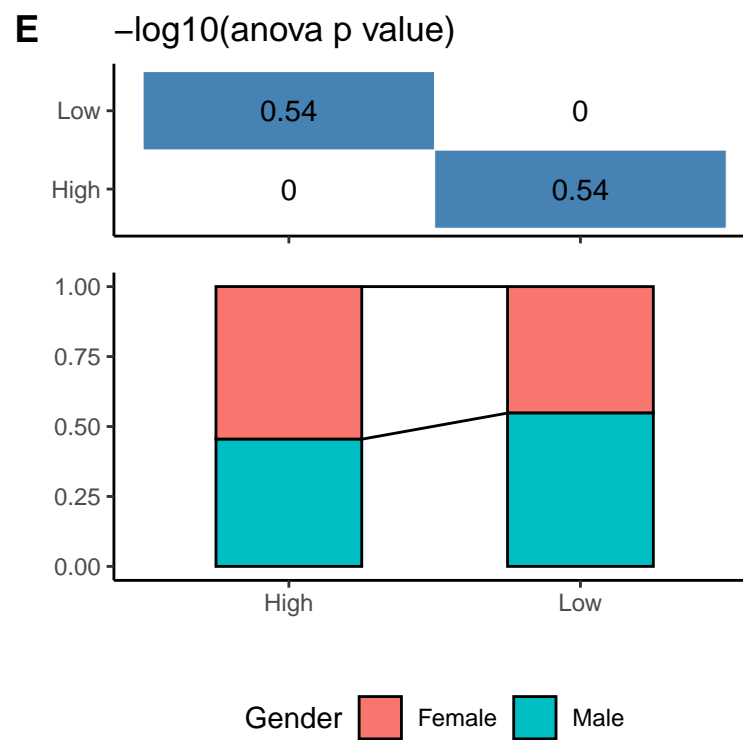

Supplement: Supplementary file 1 [file DataSheet2.PDF]

A

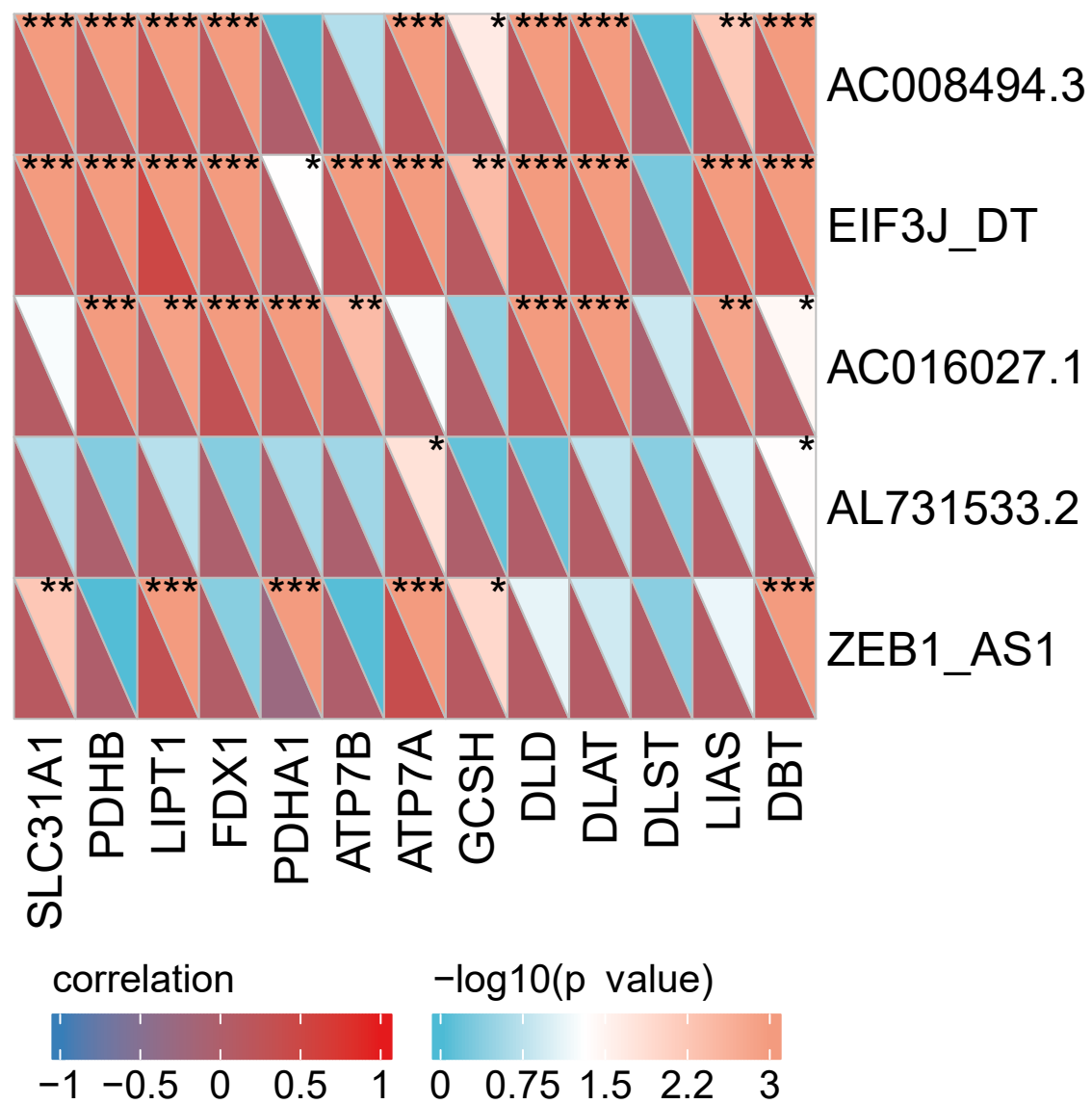

B

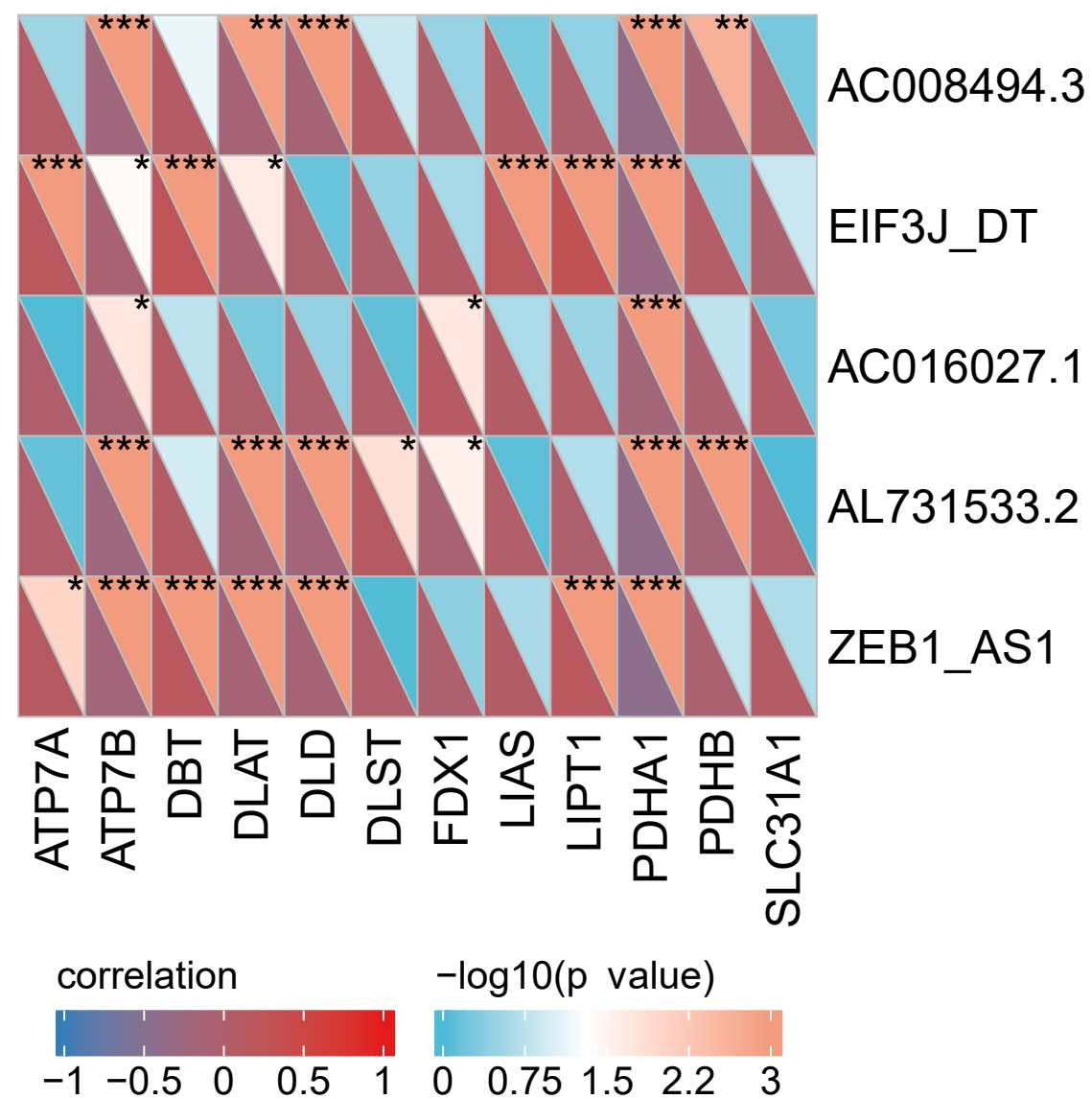

C

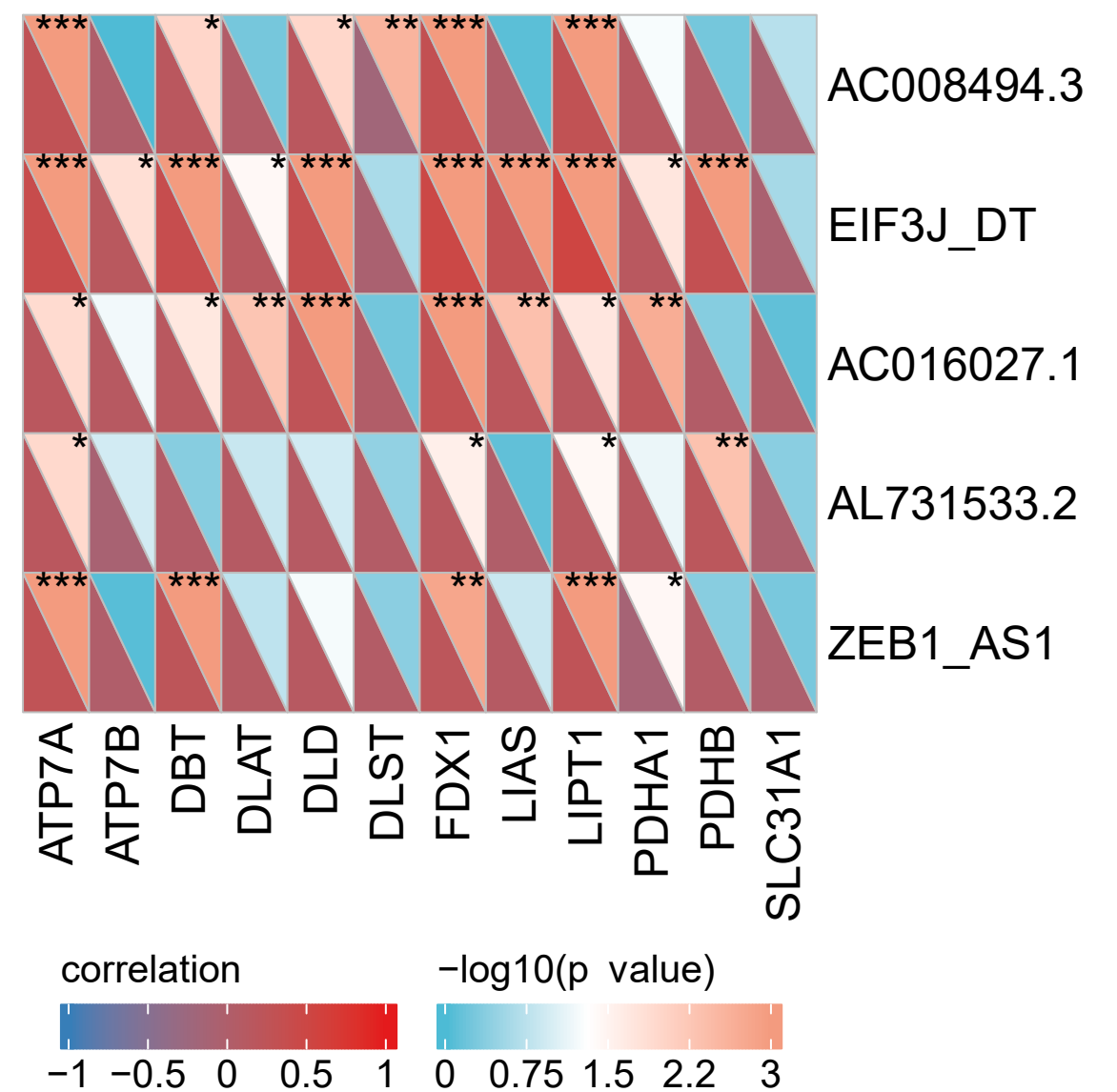

D

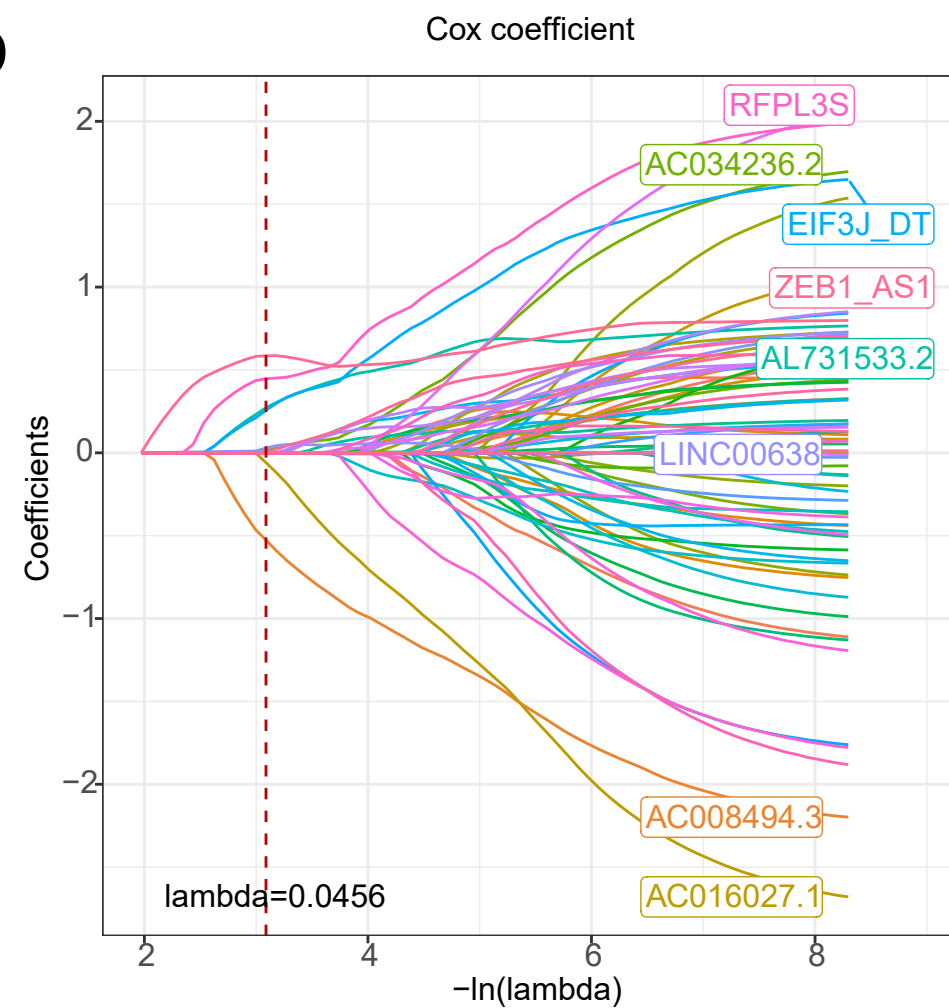

E

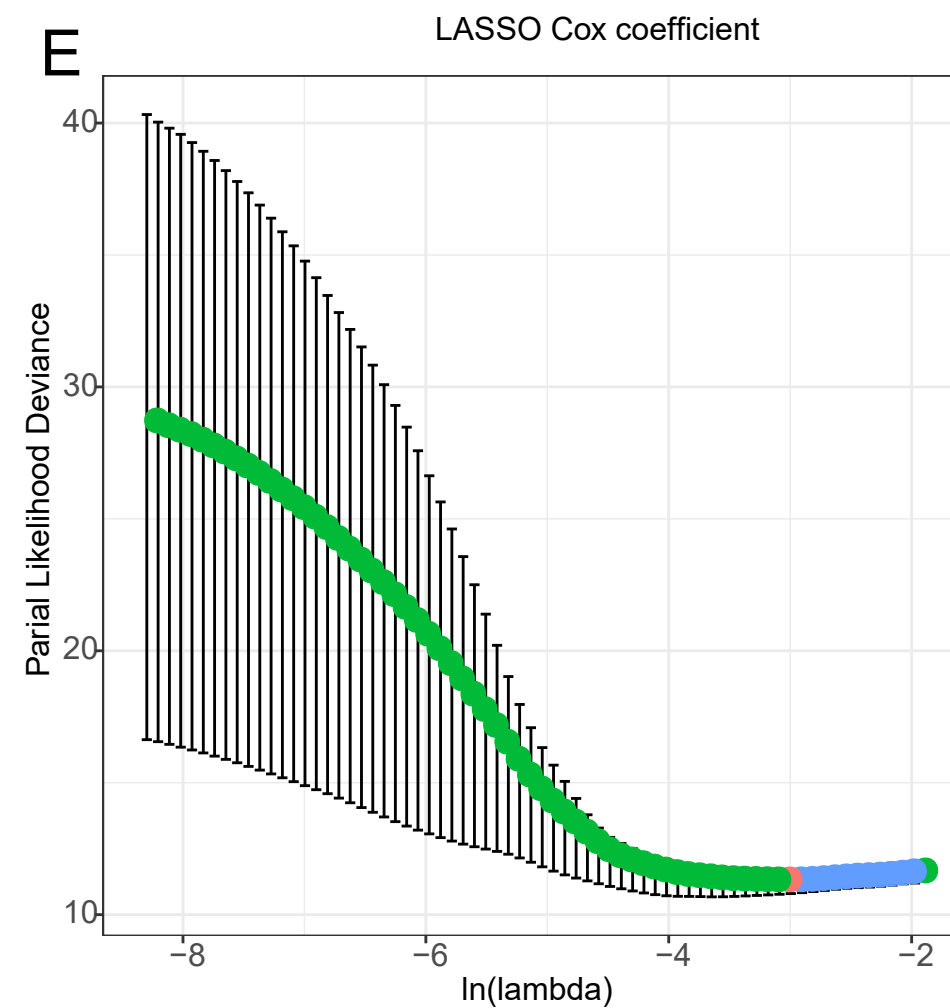

F

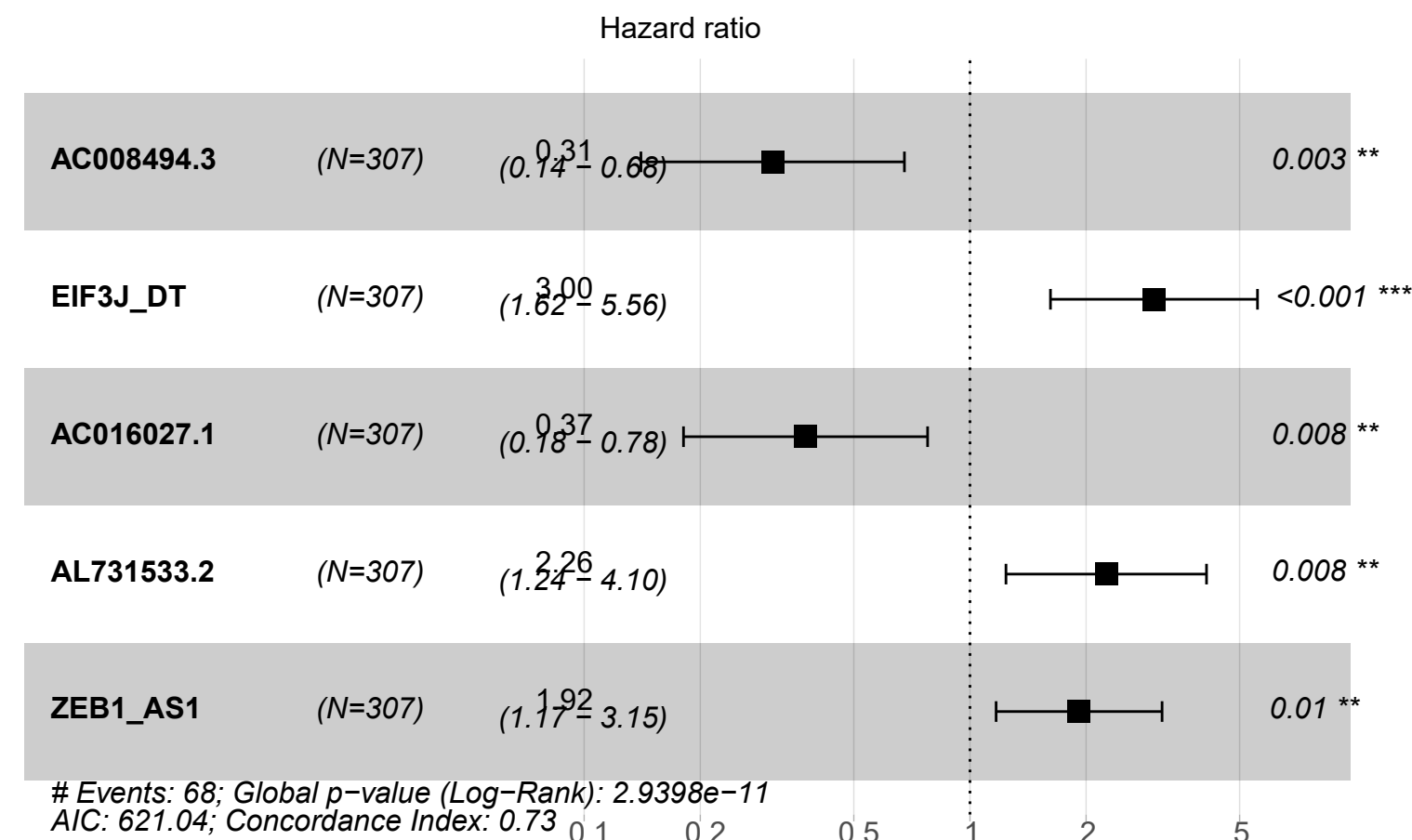

Supplement: Supplementary file 3 [file DataSheet6.PDF]

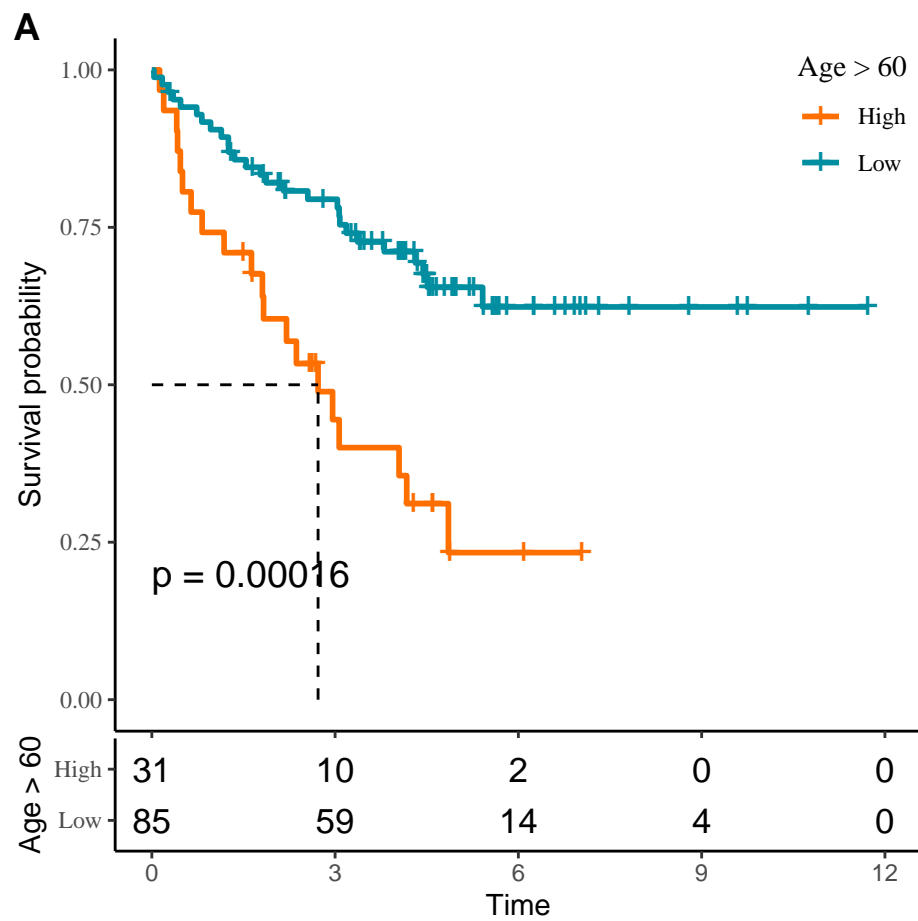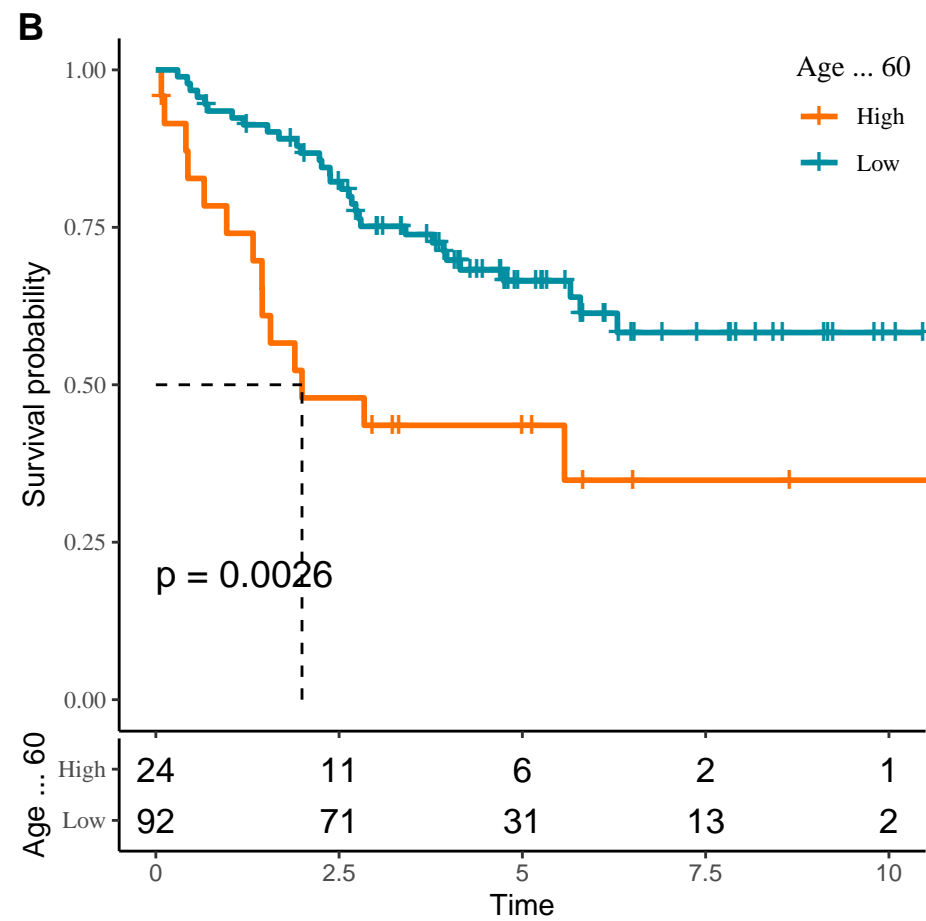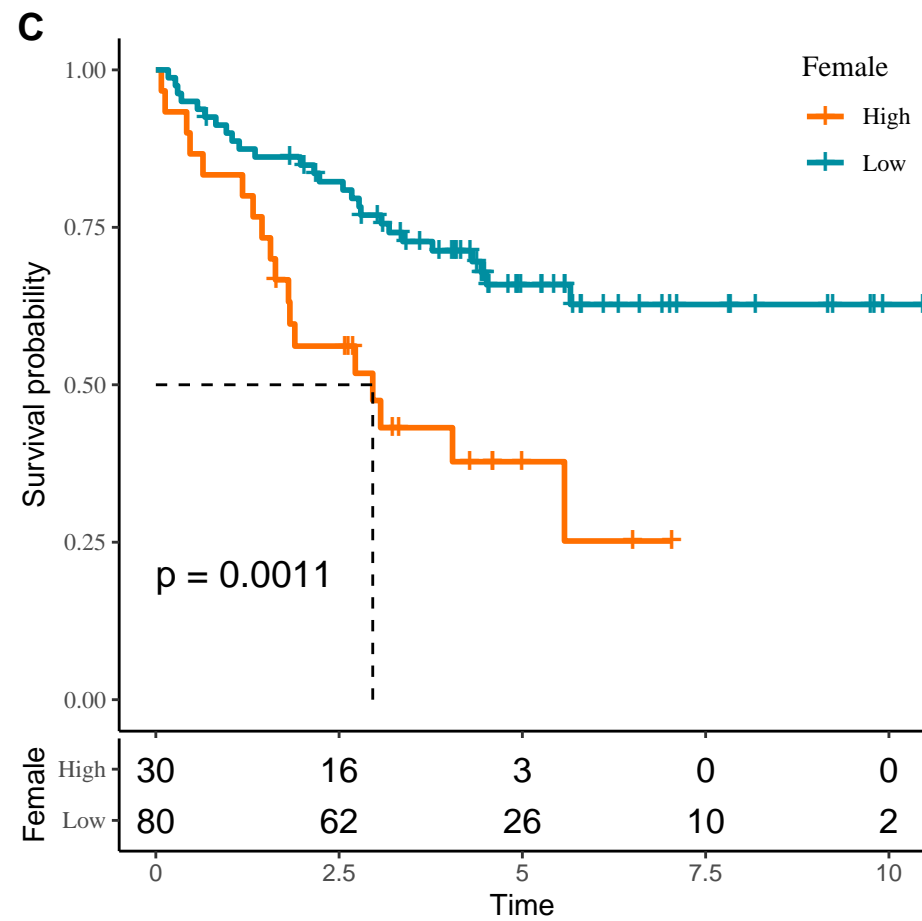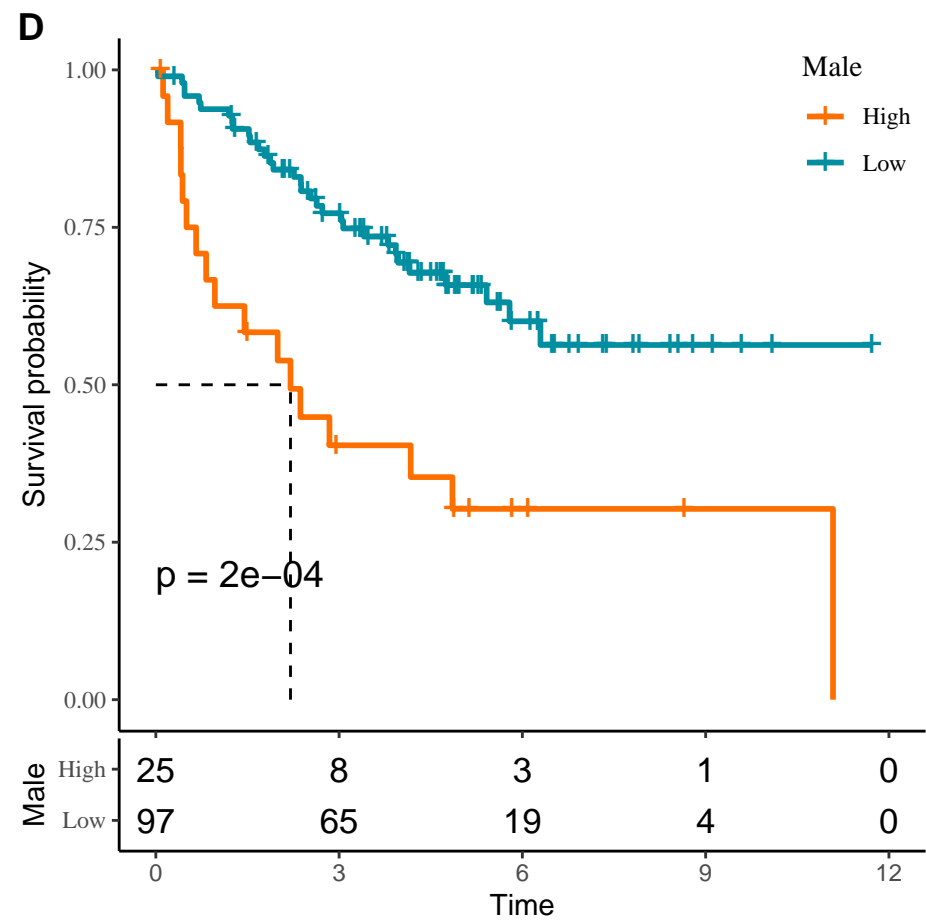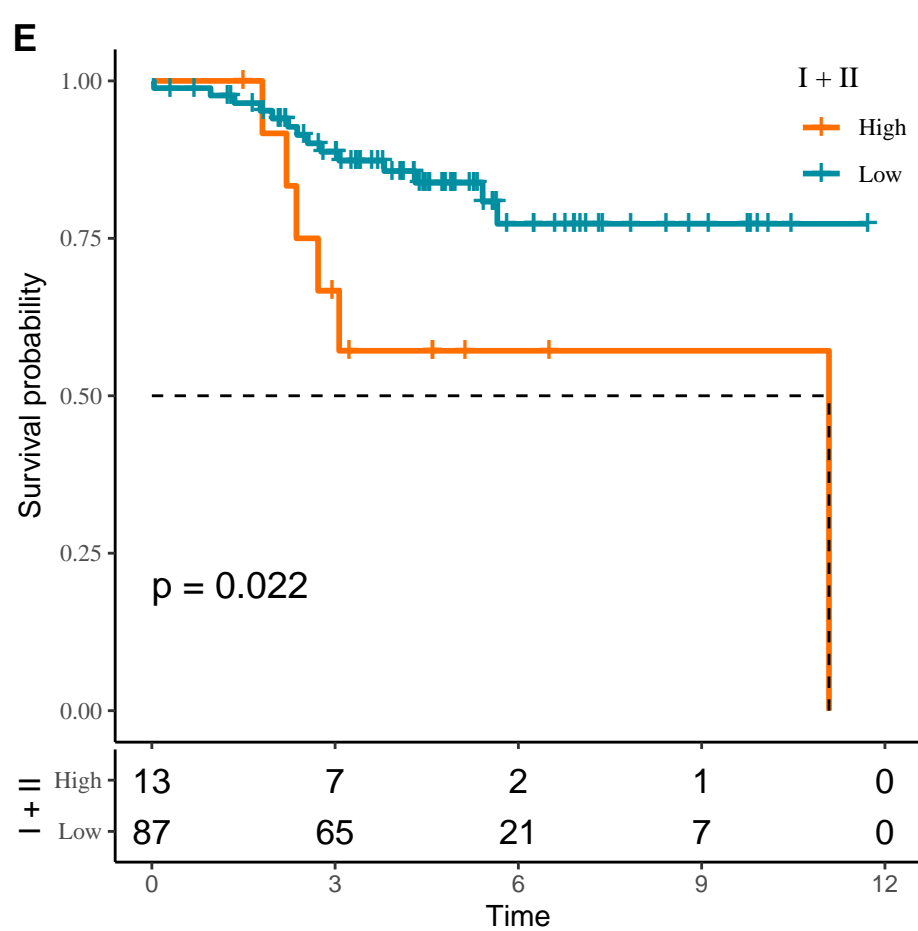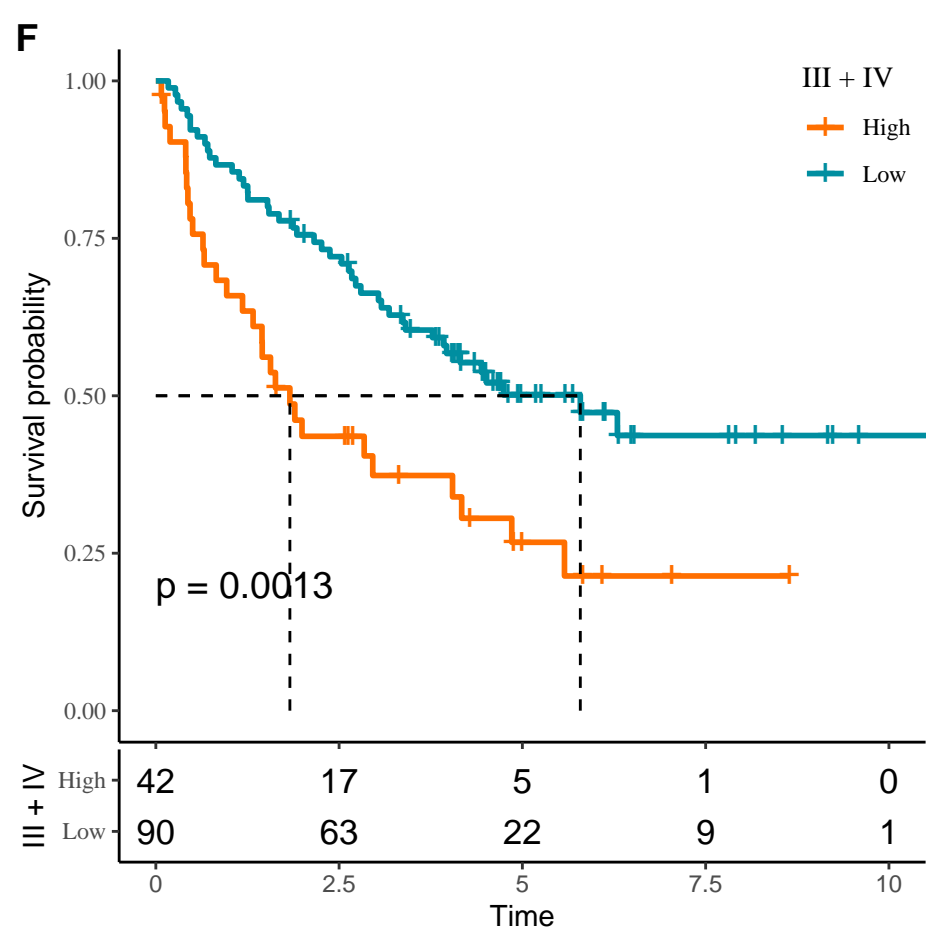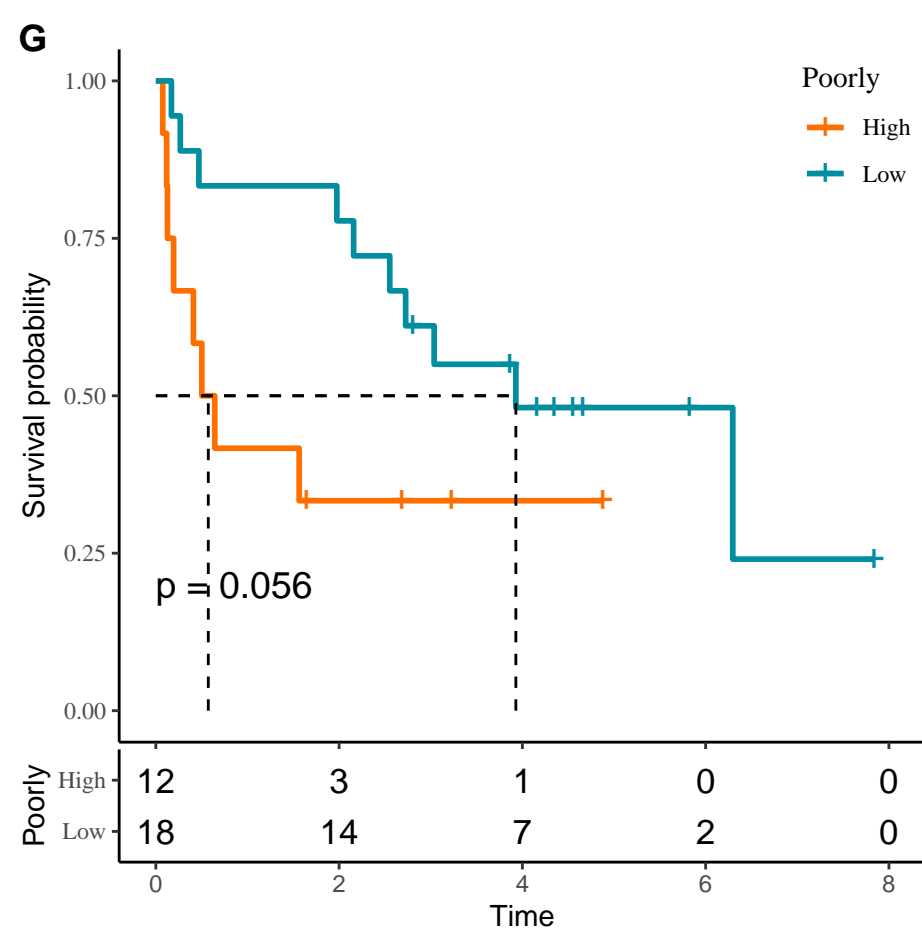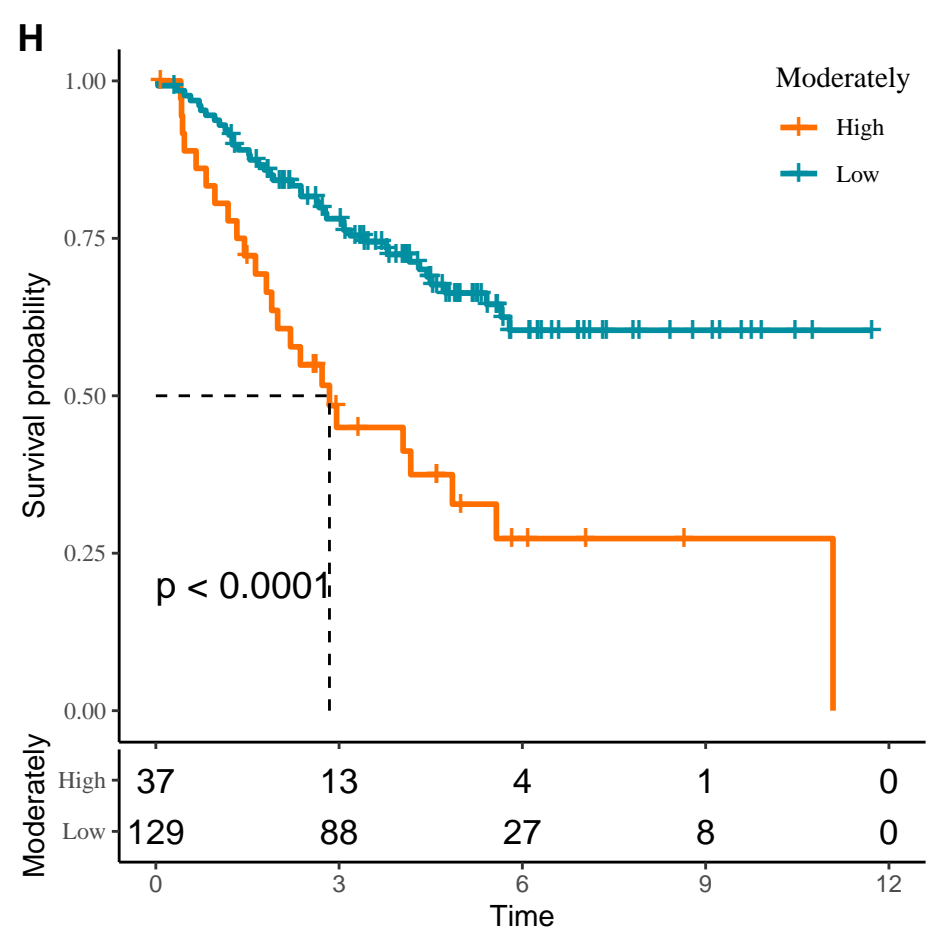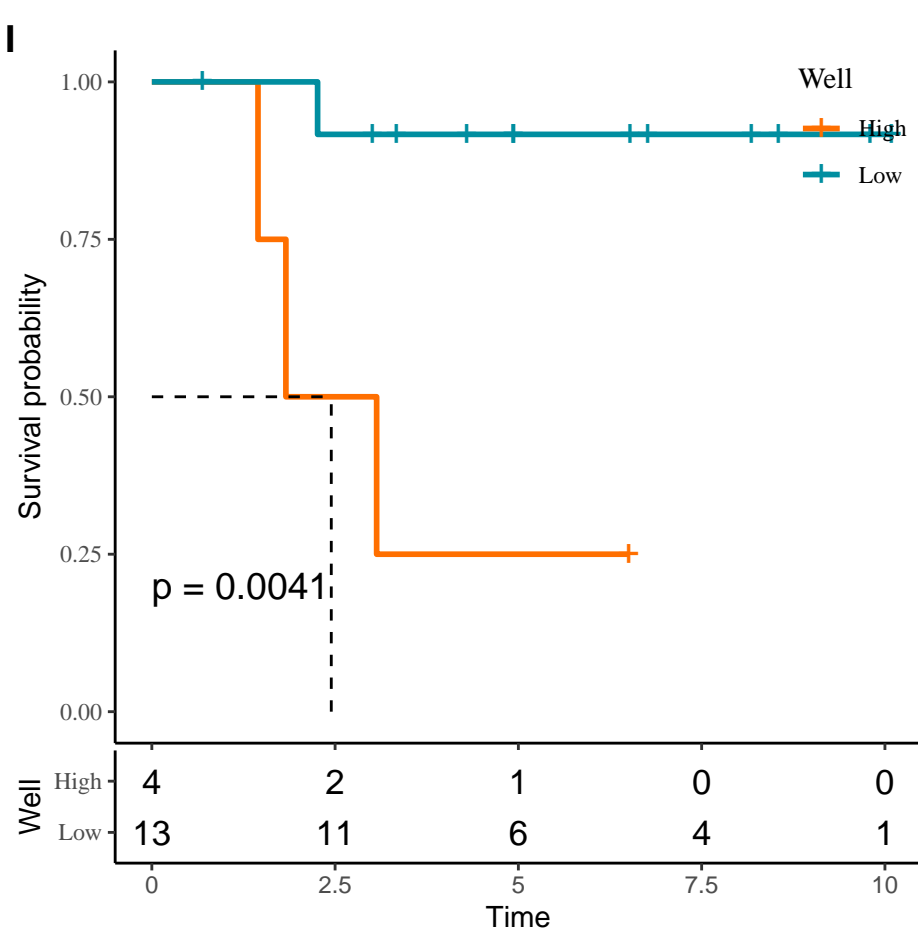

Supplement: Supplementary file 5 [file DataSheet3.PDF]

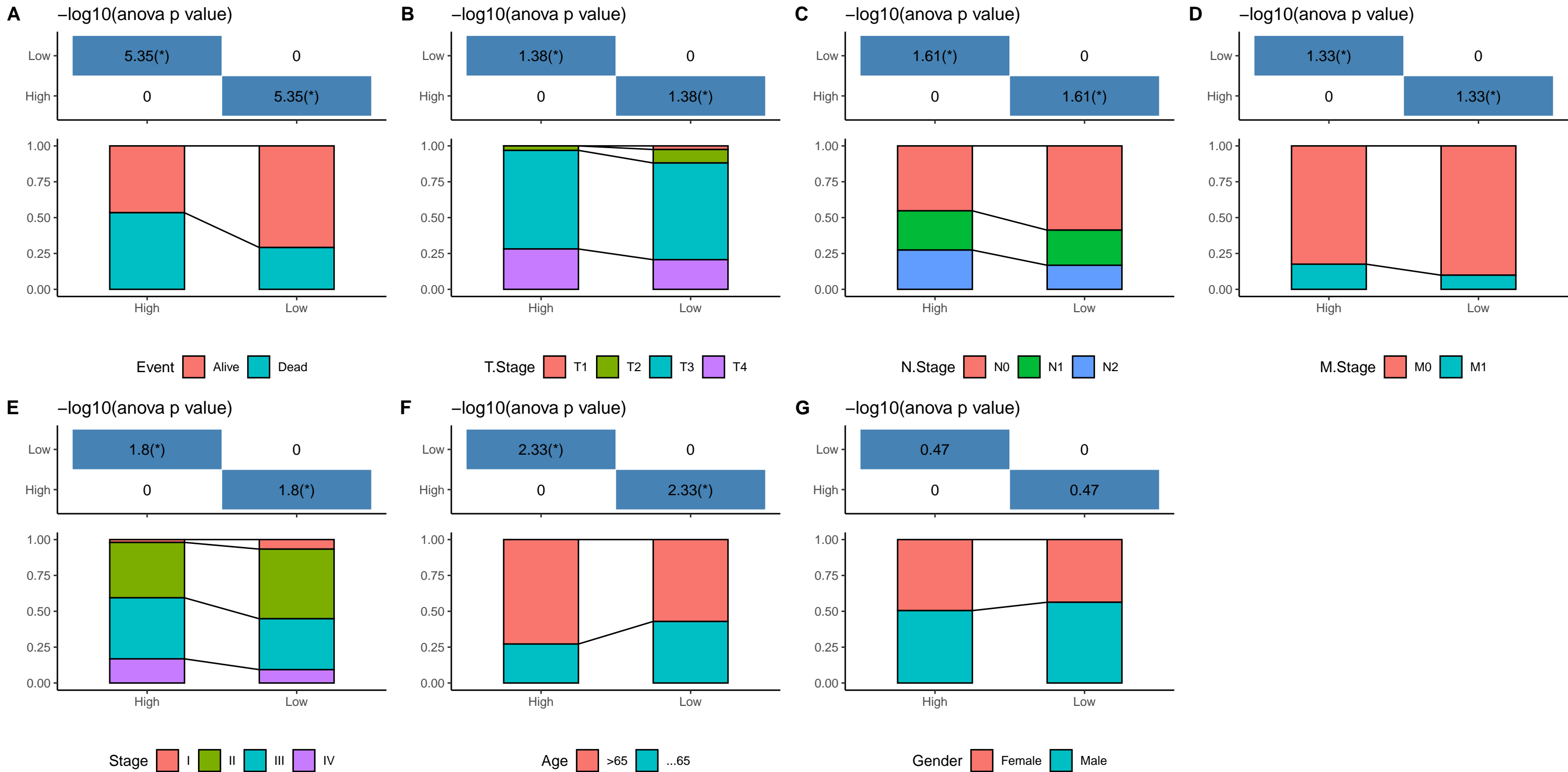

Supplement: Supplementary file 6 [file DataSheet1.PDF]

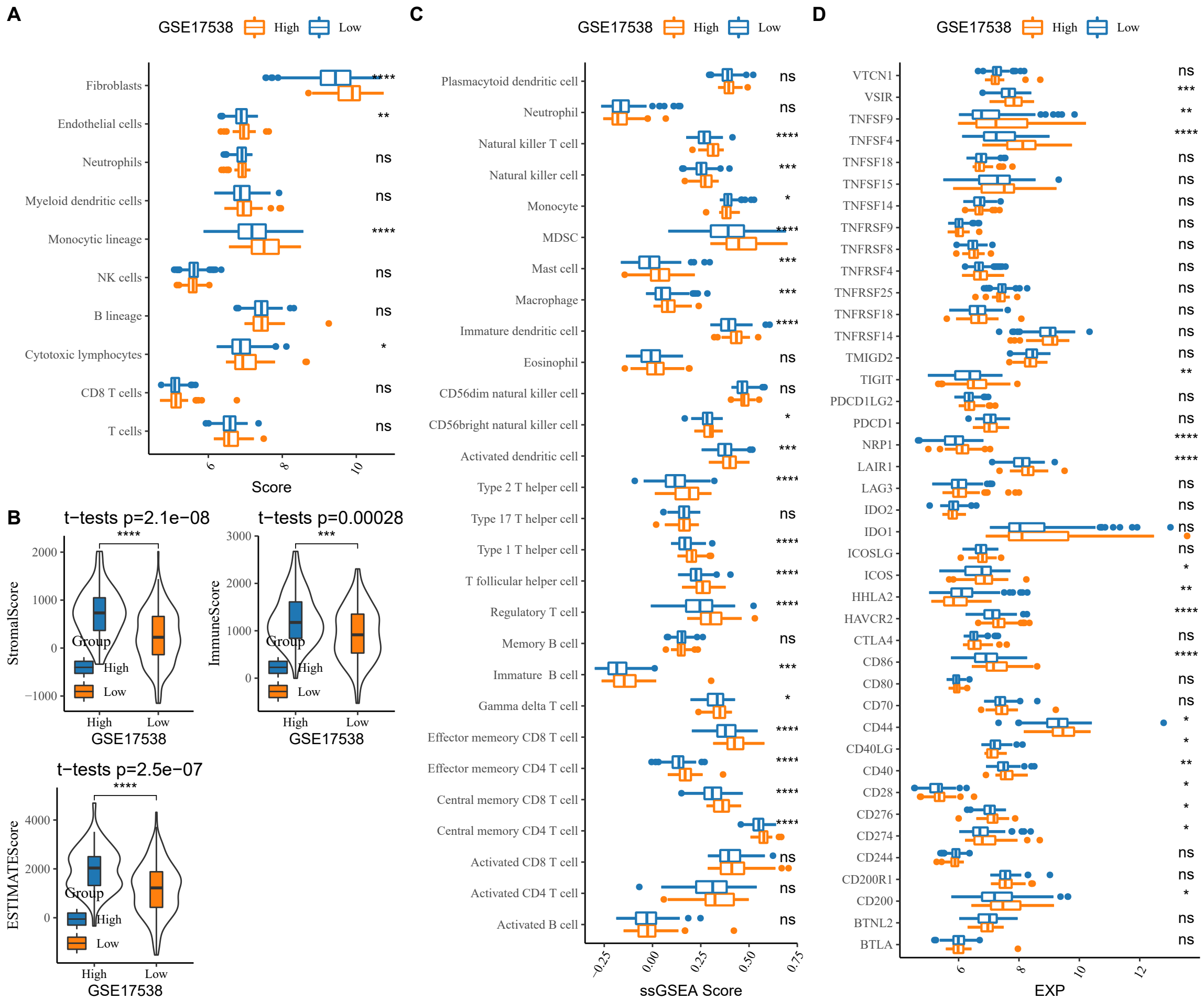

Supplement: Supplementary file 7 [file DataSheet5.PDF]
