# Supplementary material for: Based on cuproptosis-related lncRNAs, a novel prognostic signature for colon adenocarcinoma prognosis, immunotherapy, and chemotherapy response
Source: Front Pharmacol. 2023 Jun 12;14:1200054. doi: 10.3389/fphar.2023.1200054 (PMC10291194; doi:10.3389/fphar.2023.1200054)
Supplement: Supplementary file 4 [file Table1.docx]

**Supplementary figure 1: Identification of COAD cuproptosis prognosis-associated lncRNAs** (A) The correlation analysis of 5 lncRNAs and cuproptosis related genes in TCGA dataset. (B) The correlation analysis of 5 lncRNAs and cuproptosis related genes in GSE39582 dataset. (C) The correlation analysis of 5 lncRNAs and cuproptosis related genes in GSE17538 dataset. (D) Trajectory of LASSO regression coefficients (E) Confidence interval for lambda (F) Forest plot of cuproptosis prognosis-associated lncRNAs.

**Supplementary figure 2:** **Differences in clinicopathological characteristics as well as mutational characteristics among high- and low-risk groups** (A-G) Statistics of Event, T.Stage, N.Stage, M.Stage, Stage, Age, Gender information for patients in the GSE39582 cohort in the high-risk and low-risk groups, with the lower half being the proportion of cases and the upper half being statistically significant statistics.

**Supplementary figure 3: Differences in clinicopathological characteristics as well as mutational characteristics among high- and low-risk groups** (A-E) Statistics of Event, Grade, Stage, Age, Gender information for patients in the GSE17538 cohort in the high-risk and low-risk groups, with the lower half being the proportion of cases and the upper half being statistically significant statistics.

**Supplementary figure 4: K-M survival curves for high- and low-risk groups in the clinicopathological subgroups of the GSE17538 cohort** (A-B) Age subgroup (C-D) Gender subgroup (E-F) Stage subgroup (G-I) M Stage subgroup.

**Supplementary figure 5: Analysis of immune cell infiltration in TME in the TCGA-COAD cohort** (A) MCP-counter analysis (B) ESTIMATE analysis (C) ssGSEA analysis (D) Immune checkpoint gene expression.

**Supplementary figure 6: Analysis of immune cell infiltration in TME in the GSE17538 cohort** (A) MCP-counter analysis (B) ESTIMATE analysis (C) ssGSEA analysis (D) Immune checkpoint gene expression.
