# Supplementary material for: Based on cuproptosis-related lncRNAs, a novel prognostic signature for colon adenocarcinoma prognosis, immunotherapy, and chemotherapy response
Source: Front Pharmacol. 2023 Jun 12;14:1200054. doi: 10.3389/fphar.2023.1200054 (PMC10291194; doi:10.3389/fphar.2023.1200054)
Supplement: Supplementary file 2 [file DataSheet4.PDF]

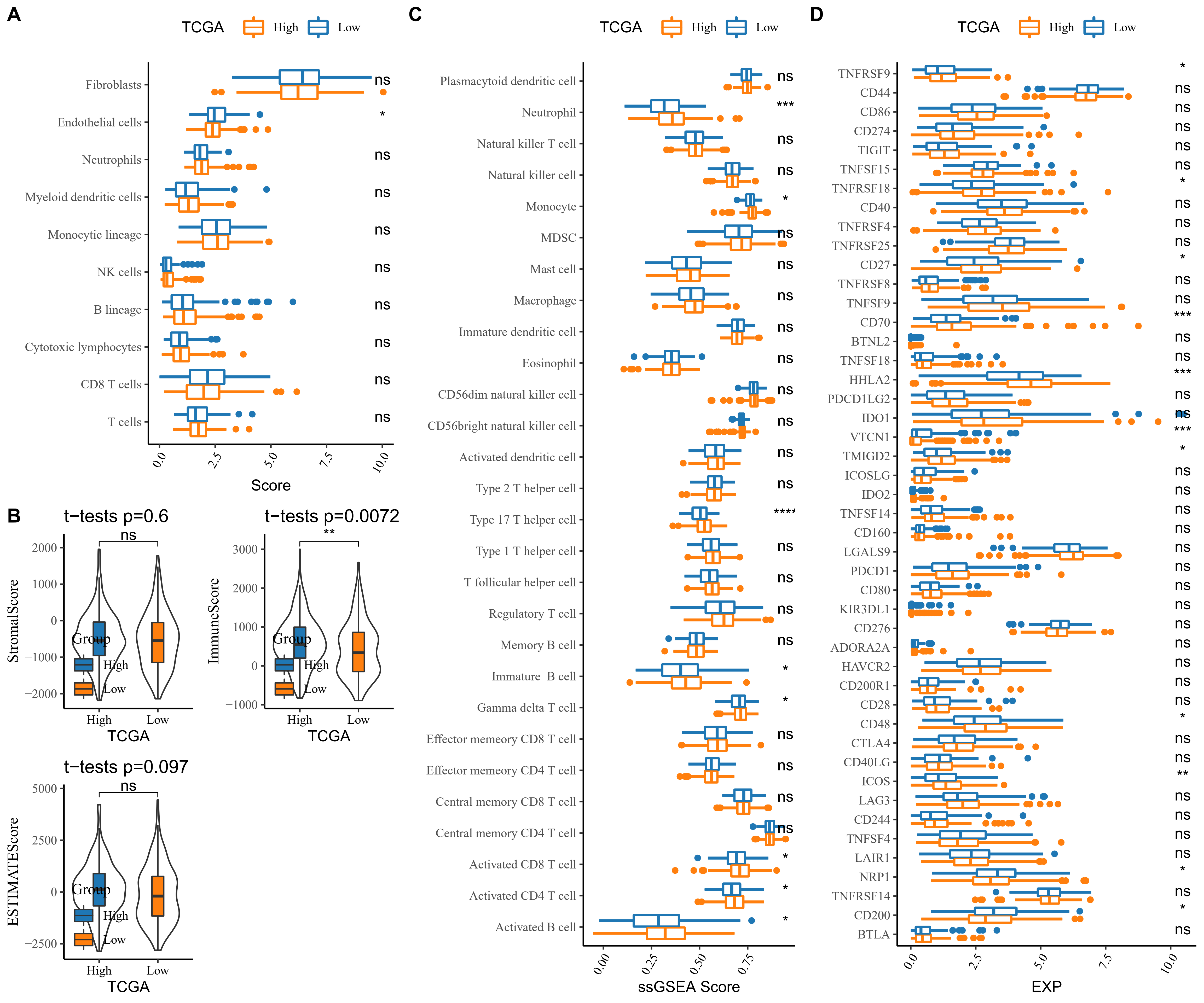
A
TCGA High Low
Fibroblasts ns
Endothelial cells *
Neutrophils ns
Myeloid dendritic cells ns
Monocytic lineage ns
NK cells ns
B lineage ns
Cytotoxic lymphocytes ns
CD8 T cells ns
T cells ns
0.0 2.5 5.0 7.5 10.0
Score
B
t−tests p=0.6
ns
StromalScore
2000 1000 0 −1000 −2000
Group High Low
High Low
TCGA
t−tests p=0.0072
**
ImmuneScore
3000 2000 1000 0 −1000
Group High Low
High Low
TCGA
t−tests p=0.097
ns
ESTIMATEScore
5000 2500 0 −2500
Group High Low
High Low
TCGA
C
TCGA High Low
Plasmacytoid dendritic cell ns
Neutrophil ***
Natural killer T cell ns
Natural killer cell ns
Monocyte *
MDSC ns
Mast cell ns
Macrophage ns
Immature dendritic cell ns
Eosinophil ns
CD56dim natural killer cell ns
CD56bright natural killer cell ns
Activated dendritic cell ns
Type 2 T helper cell ns
Type 17 T helper cell ****
Type 1 T helper cell ns
T follicular helper cell ns
Regulatory T cell ns
Memory B cell ns
Immature B cell *
Gamma delta T cell *
Effector memeory CD8 T cell ns
Effector memeory CD4 T cell ns
Central memory CD8 T cell ns
Central memory CD4 T cell ns
Activated CD8 T cell *
Activated CD4 T cell *
Activated B cell *
0.00 0.25 0.50 0.75
ssGSEA Score
D
TCGA High Low
TNFRSF9 *
CD44 ns
CD86 ns
CD274 ns
TIGIT ns
TNFSF15 ns
TNFRSF18 *
CD40 ns
TNFRSF4 ns
TNFRSF25 ns
CD27 *
TNFRSF8 ns
TNFSF9 ns
CD70 ***
BTNL2 ns
TNFSF18 ns
HHLA2 ***
PDCD1LG2 ns
IDO1 ns
VTCN1 ***
TMIGD2 *
ICOSLG ns
IDO2 ns
TNFSF14 ns
CD160 ns
LGALS9 ns
PDCD1 ns
CD80 ns
KIR3DL1 ns
CD276 ns
ADORA2A ns
HAVCR2 ns
CD200R1 ns
CD28 ns
CD48 *
CTLA4 ns
CD40LG ns
ICOS **
LAG3 ns
CD244 ns
TNFSF4 ns
LAIR1 ns
NRP1 *
TNFRSF14 ns
CD200 *
BTLA ns
0.0 2.5 5.0 7.5 10.0
EXP
